# Unique pattern of dietary adaptation in the dentition of Carnivora: its advantage and developmental origin

**DOI:** 10.1098/rspb.2016.0375

**Published:** 2016-06-15

**Authors:** Masakazu Asahara, Kazuyuki Saito, Takushi Kishida, Katsu Takahashi, Kazuhisa Bessho

**Affiliations:** 1 Primate Research Institute, Kyoto University, Inuyama, Aichi, Japan; 2 Department of Oral and Maxillofacial Surgery, Graduate School of Medicine, Kyoto Univerisity, Kyoto, Japan; 3 Wildlife Research Center, Kyoto Univerisity, Kyoto, Japan

**Keywords:** inhibitory cascade, dental morphology, *Bmp7*, Carnivora, Creodonta, Dasyuromorphia

## Abstract

Carnivora is a successful taxon in terms of dietary diversity. We investigated the dietary adaptations of carnivoran dentition and the developmental background of their dental diversity, which may have contributed to the success of the lineage. A developmental model was tested and extended to explain the unique variability and exceptional phenotypes observed in carnivoran dentition. Carnivorous mammalian orders exhibited two distinct patterns of dietary adaptation in molars and only Carnivora evolved novel variability, exhibiting a high correlation between relative molar size and the shape of the first molar. Studies of *Bmp7*-hetero-deficient mice, which may exhibit lower *Bmp7* expression, suggested that *Bmp7* has pleiotropic effects on these two dental traits. Its effects are consistent with the pattern of dietary adaptation observed in Carnivora, but not that observed in other carnivorous mammals. A molecular evolutionary analysis revealed that *Bmp7* sequence evolved by natural selection during ursid evolution, suggesting that it plays an evolutionary role in the variation of carnivoran dentition. Using mouse experiments and a molecular evolutionary analysis, we extrapolated the causal mechanism of the hitherto enigmatic ursid dentition (larger M_2_ than M_1_ and M_3_). Our results demonstrate how carnivorans acquired novel dental variability that benefits their dietary divergence.

## 1. Introduction

A major goal of evolutionary biology is to explain why some taxa outcompete others during evolution. Variability (i.e. patterns of variation that can be generated) is a critical factor in evolution [1,2]. The route, speed and potential for evolution (evolvability) are biased by patterns of variation (e.g. covariation between traits) [1,2]. Therefore, evolutionary pathways and phenotypic diversity among taxa or populations are often consistent with the pattern of variation among individuals within a population (e.g. [1–3]). Evolutionary developmental (evo–devo) biology integrates evolutionary, adaptational and developmental approaches as well as the developmental basis of variability or covariation of traits [4]. However, evo–devo studies in non-model organisms and fossil taxa are beset with difficulties (e.g. [5,6]). In this study, we provide a basis for understanding the developmental origins of the variability or covariation of traits that contribute to various dietary adaptations and long-time lineage success using an integrated approach.

Three mammalian orders exhibit carnivorous dietary adaptations: the extant orders Carnivora and Dasyuromorphia and the extinct Creodonta [7,8]. Of these orders, Carnivora is the most long-lived and successful mammalian taxon in terms of dietary divergence [7,8]. For example, Carnivora and Creodonta, which are considered sister orders, were competitors during the Palaeogene period [9,10], but carnivorans (the order Carnivora) were ultimately more successful. The observed diversity and dietary divergence of these two orders are often attributed to an evolutionarily novel structure, namely flesh-shearing teeth known as carnassials [7,11,12]. However, the carnassials in these orders are derived from different teeth [7,11,12]. Carnassials evolved from premolars and mesial molars (e.g. P^4^ and M_1_) in carnivorans but distal molars (e.g. M^1^ and M_2_ or M^2^ and M_3_) in creodonts [7,11,12]. These established differences were present at the origin of these taxa [11]. In addition, the present success of Carnivora is owing, to a large extent, to their evolutionary versatility; that is, they possess grinding teeth in addition to carnassials [7,8]. However, how these differences affected their success over creodonts has not been evaluated in functional morphology and evo–devo frameworks.

**Figure 1.**
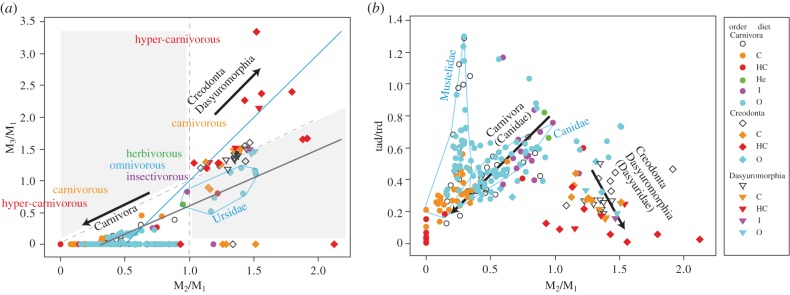
Variation and dietary adaptation pattern of lower molars in three mammalian orders. (*a*) Plots of M_2_/M_1_ versus M_3_/M_1_. Each data point indicates one species (electronic supplementary material, table S1); the shape indicates the taxon (circle, Carnivora; diamond, Creodonta; triangle, Dasyuromorphia) and the colour indicates the diet (orange, carnivorous; red, hyper-carnivorous; green, herbivorous; purple, insectivorous; blue, omnivorous). The blue line indicates the original regression line in the IC model [13]. The black line indicates the regression of the species in Carnivora that have three lower molars. The grey areas indicate patterns that cannot be explained by the IC model (M_1_ < M_2_ > M_3_ or M_1_ > M_2_ < M_3_) [14]. Omnivorous and insectivorous species tend to have equal-sized molars. Carnivorous carnivorans tend to have larger M_1_, but carnivorous creodonts and dasyuromorphians tend to have larger mesial molars (smaller M_1_). Arrowed lines indicate two types of carnivorous adaptations among three orders. (*b*) Plots of M_2_/M_1_ versus tad/trd. Carnivorous species tend to have relatively larger trigonids relative to omnivorous and insectivorous species in all three orders.

A developmental model called the inhibitory cascade (IC) model proposes that the relative size of the lower molars is governed by the balance of inhibitory molecules secreted by the M_1_ tooth germ and activation molecules from the mesenchyme [13]. Accordingly, relative molar sizes vary from M_1_ > M_2_ > M_3_ to M_1_ = M_2_ = M_3_ to M_1_ < M_2_ < M_3_ along a particular regression line in the M_2_/M_1_ versus M_3_/M_1_ morphospace [13] (figure 1). This model can explain interspecific variation in many mammalian groups except for several bear, horse and vole species [13–21]. However, some taxa, such as canids (i.e. Canidae, Carnivora), exhibit unique patterns with small slopes; the slope of the M_2_/M_1_ versus M_3_/M_1_ regression in canids (0.45) is smaller than that indicated by the IC model in murines (2.0) [13]. The correlation between M_2_/M_1_ and M_3_/M_1_ indicates that they basically fit the IC model (the inhibition/activation mechanism affects both M_2_/M_1_ and M_3_/M_1_) [15]. However, ursids (i.e. Ursidae, Carnivora) exhibit M_1_ < M_2_ > M_3_, which could not be explained by the model [14]. Several members of equines (horses) and arvicolines (voles) also exhibit M_1_ < M_2_ > M_3_ or M_1_ > M_2_ < M_3_, which cannot be explained by the model [14,16,17]. The interspecific slope for other carnivorous mammals, the causes underlying the differences in slopes from the IC model and the exceptional dental pattern observed in ursids remain unclear.

Previous studies have reported varying IC patterns (slope of M_2_/M_1_ versus M_3_/M_1_ regression) among mammalian taxa (slope range: 0.45–3.269 [13,15–20]). The smallest slope was reported in Canidae, Carnivora [15], while the original experiment by Kavanagh *et al*. [13] resulted in slope = 2. These low slopes can be generated by a reduction in the M_3_/M_1_ ratio or an increase of the M_2_/M_1_ ratio. A previous study has suggested that particular signalling molecules (e.g. a diffusible inhibitor or its antagonist) with low diffusibilities can result in small slopes because they may affect only M_2_/M_1_ and not M_3_/M_1_ [15]. However, this hypothesis has not been tested.

The relative molar sizes (i.e. the relative sizes of M_1_, M_2_ and M_3_) are reliable indicators of diet in comparative analyses of murines [13], canids [15,22] and other mammalian orders [20]. This property is at least partly due to a high evolvability; changes in parameters underlying a single developmental mechanism (e.g. IC) can generate varying molar sizes [13]. According to previous ecomorphological studies, the proportions of shearing and grinding regions within a molar row reflect diet in mammals; the shearing function is important for carnivorous diets and the grinding function for omnivorous diets [23–25]. In Carnivora, the trigonid of the M_1_ functions in shearing flesh and the talonids of the M_1_ and the other molars function in crushing various objects [8,11,12]. In Creodonta and Dasyuromorphia, the trigonids of all molars retain the shearing function, but that of the carnassial is most prominent [8,11,12]. Therefore, relative molar size and carnassial shape (relative trigonid size) play important and independent roles in dietary adaptations in mammals. However, the adaptive evolution of these traits has not been investigated separately or integrated into an evo–devo framework.

Evolutionary pathways are constrained by the phenotypic variability and covariation of traits [1,2]. In dental morphology, various dental traits can change simultaneously when particular signalling molecules are modified [26]. If various dental traits are modified simultaneously (generating covariation among the traits) to adapt to a particular diet via pleiotropic effects, the evolutionary pathway can be considered canalized or constrained by the developmental mechanism. In this manuscript, the direction of morphological changes involved in the evolution of particular diets that are shared among taxa is referred as an ‘adaptation pattern’. If an adaptation pattern observed in a particular taxon relates to (or correlates to) variability or covariation caused by a developmental mechanism, the constraint can benefit the taxon, perhaps enabling easier and rapid evolution. For example, in a particular taxon, if a change in only one signalling molecule during development generates a set of phenotypic changes that contribute to dietary adaptation, this developmental mechanism can be considered advantageous to the taxon.

In this context, the investigation of pleiotropic effects related to dental traits is crucial for determining the developmental mechanism that constrains or promotes evolution in a particular taxon. In other words, the developmental mechanism that shapes the adaptation pattern with respect to competitors can be crucial. We focused on *Usag-1* (uterine sensitization-associated gene-1, also known as ectodin or Sostdc1/Sclerostin domain-containing protein 1) and *Bmp7* (bone morphogenetic protein 7). USAG-1 was treated as a diffusible inhibitor in the IC model by Kavanagh *et al*. [13] and USAG-1-deficient mice have supernumerary molars [27]. BMP7 is a USAG-1 antagonist and is expressed during dental development [28]. There is support for the homology of each cusp (e.g. protocone), talonid and trigonid between rodents and other mammals, including carnivorans [29–33]. Therefore, we used genetically modified mice to investigate the phenotypes affected by these two molecules.

After we identified candidate molecules associated with the evolved phenotypes, we examined their molecular evolution to confirm whether these molecules evolved among the targeted taxa. Along the lineages in which we observed significant molecular evolution, we also investigated phenotypic evolution to examine the relationship between phenotypic and molecular evolution.

In this study, we examined the advantages of Carnivora relative to other taxa by investigating (i) the variability in molars, including IC patterns (slope of the M_2_/M_1_ versus M_3_/M_1_ regression) and carnassial shape, in carnivorous mammals, (ii) patterns of dietary adaptation in relative molar size and carnassial shape in these mammals, (iii) phenotypes of USAG-1 or BMP7 hetero-deficient mice, and (iv) molecular and phenotypic evolution along ursid lineages. Finally, we show a pattern of covariation between carnassial shape and relative molar size, which represents the developmental basis that promoted dietary diversification of the Carnivora.

## 2. Material and methods

### (a) Species

Mandible specimens of 258 living and fossil species belonging to Carnivora, Creodonta and Dasyuromorphia were examined. Measurement data are summarized in the electronic supplementary material, text S1 and table S1.

### (b) Dietary categories

Diets were categorized as hyper-carnivorous (HC; these animals have diets largely consisting of mammals or birds, or taxa larger than themselves with respect to body mass), carnivorous (C; these animals are primarily flesh-eaters), omnivorous (O; these animals eat various foods, with neither flesh nor insects comprising more than 50% of their diet), insectivorous (I; these animals are primarily insect-eaters) or herbivorous (He; these animals are primarily leaf-eaters). For both extant and fossil species, dietary information was obtained from the literature (electronic supplementary material, text S2 and table S1).

### (c) Morphological analysis

**Figure 2.**
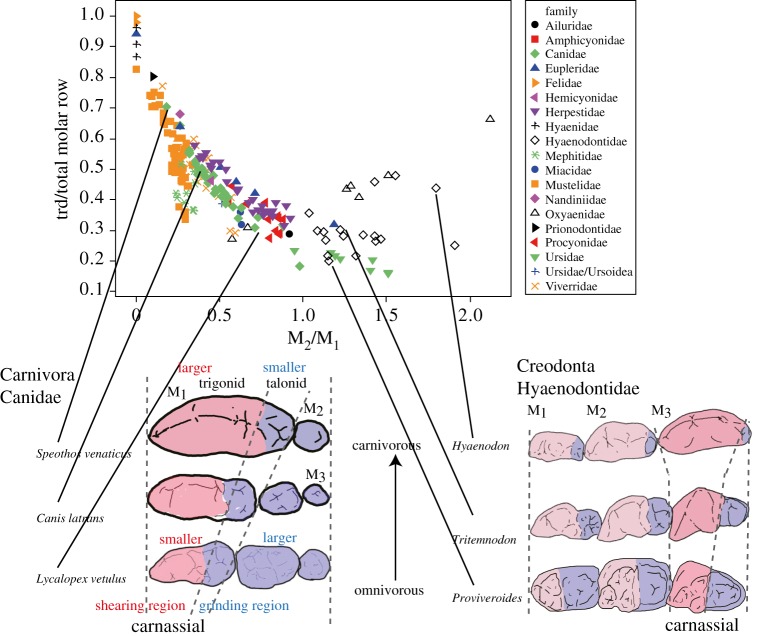
Variability in the relative trigonid size of the carnassial in relation to the total molar row in Carnivora and Creodonta. Plot of trigonid size of the carnassial (trd) divided by total molar row, with illustrations of diversity in molar rows among Canidae (Carnivora) and Hyaenodontidae (Creodonta). Trigonid of the carnassial is shown in deep red. The relative carnassial-trigonid size varied more highly in Carnivora than in Creodonta.

The lower molar rows were photographed from the occlusal perspective, and the projected areas of three molars (M_1_, M_2_ and M_3_), as well as the trigonids (trd) and talonids (tad) of the carnassials (M_1_ in Carnivora, M_3_ or M_2_ in Creodonta and M_4_ in Dasyuromorphia) were measured using ImageJ (NIH, Bethesda, MD, USA). To investigate the application of the IC model in carnivorous mammals, relative molar sizes (i.e. M_2_/M_1_ and M_3_/M_1_) were measured. In addition, carnassial shape was measured as the ratio of the trigonid to talonid (tad/trd) to test the correlation between IC and tooth shape (tad/trd). The ridge was considered the border between the trigonid and talonid areas of the carnassial (M_1_ for Carnivora, M_2_ or M_3_ for Creodonta and M_4_ for Dasyuromorphia; figure 2). In addition, the ratio of carnassial-trigonid size to that of the total molar row was compared between Carnivora and Creodonta to visualize the variability in the relative shearing region among molar rows. Among M_2_/M_1_, M_3_/M_1_ and tad/trd, reduced major axis (RMA) regressions were performed using PAST [34]. RMA was chosen based on our hypothesis that the ratios are explained by another factor (i.e. inhibition/activation) and according to the design of previous studies [13,15].

### (d) Macroevolutionary analyses and phylogenetic analysis of variance

An RMA regression analysis of M_3_/M_1_ and tad/trd on M_2_/M_1_ was performed. Differences in M_2_/M_1_ and tad/trd between dietary categories were tested by analysis of variance (ANOVA) with Tukey's tests as the most common comparison method; additionally, a phylogenetic ANOVA was performed to avoid phylogenetic biases.

Phylogenetic ANOVA [35] were performed using phytools in R [36] with 1000 simulation replicates and a Bonferroni–Holm correction for post hoc tests. Branch lengths were estimated using methods described by Pagel [37]. The phylogenetic tree used for the phylogenetic ANOVA was obtained from previous studies (described in the electronic supplementary material, text S3 and figures S3–S4). Owing to the lower availability of phylogenetic information, fewer species were used for phylogenetic ANOVA than ANOVA/Tukey's tests.

### (e) Production of *Usag-1*- and *Bmp7*-deficient mice

*Bmp7*- and *Usag-1*-deficient mice were produced from previously generated lines [38]; *Usag-1*-deficient mice had the C57Bl6/J background and *Bmp7*-deficient mice had the ICR background. Double-knockout mice were generated by crossing two mouse lines. To circumvent the effect of background, only F2 progeny were analysed. To avoid the use of specimens possessing supernumerary and/or fused teeth [27], heterozygous (Het: hetero-deficient) and wild-type (WT) F2 mice were used for the analysis.

### (f) Morphological comparison of genotypes

**Figure 3.**
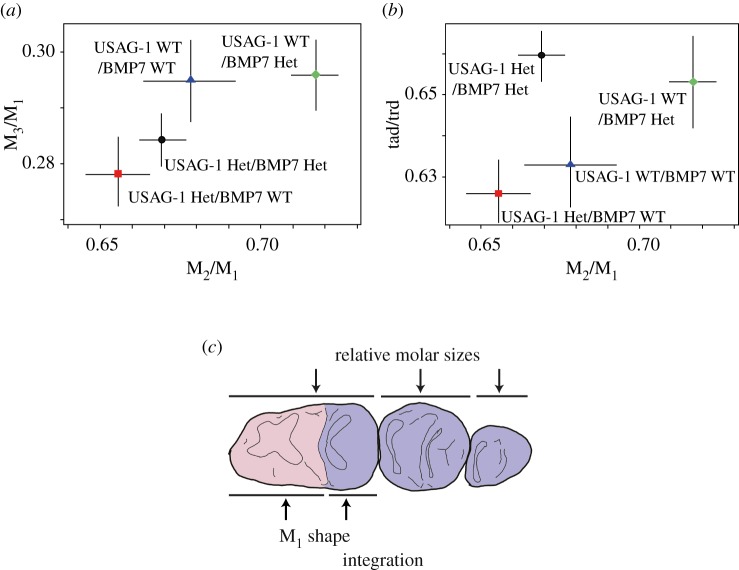
Difference in relative molar sizes and M_1_ shape (relative trigonid and talonid size) between mice of different *Usag-1* and *Bmp7* genotypes. (*a*) Plots of M_2_/M_1_ versus M_3_/M_1_. Het, hetero-deficient; WT, wild-type. Plots are averages of each genotype and two-dimensional bars indicate the standard error. (*b*) Plots of M_2_/M_1_ versus tad/trd. Our results suggest that *Bmp7* Het (i.e. a decrease in BMP7) generates larger M_2_/M_1_ and tad/trd scores. (*c*) Schematic illustration of mouse dentition for the integration of relative molar sizes and M_1_ shape caused by BMP7.

For the dry skulls of F2 mice, the lower molar rows from the occlusal perspective were photographed, and the projected areas of the three molars (M_1_, M_2_ and M_3_) [39] as well as the trigonids (trd) and talonids (tad) of the M_1_ (the segmentation of the trigonid and talonid is shown in figure 3*c*) were measured. The general linear model (GLM) was applied to determine the effects of USAG-1, BMP7 and their statistical interactions based on the results of the double-deficient mouse phenotypes. After applying the Anderson–Daring normality test, the effects of USAG-1, BMP7 and their interaction on M_2_/M_1_, M_3_/M_1_ and tad/trd were examined using GLM implemented in Minitab 14 (Minitab, Inc., PA, USA).

### (g) Molecular evolution of *BMP*7

The nucleotide sequences of the *Bmp7* genes of all available carnivoran species and appropriate outgroups were retrieved from public databases, and non-synonymous and synonymous substitutions along the branches of the phylogenetic tree were calculated. BMP7 consists of two distinctive domains, the pro-domain and the mature-domain [40–42], which were analysed separately. Details of this analysis are provided in the electronic supplementary material, text S4).

## 3. Results

### (a) Inhibitory cascade pattern and carnassial shape in carnivorous mammals

In the M_2_/M_1_ and M_3_/M_1_ morphospace, the phenotypes of most species could be explained by the IC model [14], except for ursids and some creodonts (figure 1*a*; electronic supplementary material, table S1). In addition, there was a positive correlation between M_2_/M_1_ and M_3_/M_1_ in all families (*p* < 0.05), also supporting the IC model; that is, the inhibition/activation mechanism affects both M_2_ and M_3_ [15] (figure 1*a*,*b*; electronic supplementary material, tables S2–S3). For most families in Carnivora, there was a correlation between M_2_/M_1_ and tad/trd (*p* < 0.05 for all families with more than 12 species), indicating a relationship between the IC and carnassial shape (tad/trd), but this relationship was not detected in Creodonta or Dasyuromorphia (*p* > 0.05; figure 1*a*,*b*; electronic supplementary material, tables S2–S3). The slopes of the regression of M_2_/M_1_ and M_3_/M_1_ in carnivorans (slope: 0.45 in Canidae species with three molars, 1.08 in Ursidae and 0.90 in all carnivoran species with three molars) were smaller than those of other orders (slope range for Creodonta and Dasyuromorphia: 2.01–2.57; electronic supplementary material, table S2).

### (b) Adaptation patterns in carnivorous mammals

Among all Carnivora, the M_2_/M_1_ and tad/trd scores tended to increase gradually from HC to C, O and I species (figure 1*a*,*b*).

The variation in these scores for carnivoran families is shown in the electronic supplementary material, figure S1a,b. Within these families, we compared the M_2_/M_1_ and tad/trd scores among dietary categories. Based on family-level comparisons, the average M_2_/M_1_ and tad/trd scores for each dietary category increased from HC, C and O to I species (electronic supplementary material, figure S1c,d). According to ANOVA with Tukey's tests, the effect of diet was not significant for several families, but was significant for families that contained sufficient numbers of species in each dietary category (such as Canidae and Mustelidae; electronic supplementary material, table S4).

In the phylogenetic ANOVA, the effect of diet was significant for all Carnivora when the effects of phylogeny were removed (*p* = 0.001; electronic supplementary material, table S4). In addition, either M_2_/M_1_ or tad/trd was significantly different for all within-family comparisons, except for Procyonidae, in which all species were categorized as being omnivorous or insectivorous (electronic supplementary material, table S4). Therefore, we concluded that the evolution of the relative molar sizes and relative size of the trigonid and talonid reflect diet in Carnivora.

According to the results of the ANOVA with Tukey's test and phylogenetic ANOVA for Creodonta, the effect of diet on M_2_/M_1_ was significant for Oxyaenidae, and the effect of diet on tad/trd was significant for Hyaenodontidae and Oxyaenidae (electronic supplementary material, table S4). The evolutionary direction of M_2_/M_1_ in Oxyaenidae was opposite to that of carnivorans; M_2_/M_1_ scores increased from I, O, C to HC species (electronic supplementary material, figure S2c). Such patterns of carnivorous evolution were also observed in Hyaenodontidae. That is, a more carnivorous subfamily Hyainailourinae, derived from Proviverrinae [43], presented larger distal molars (electronic supplementary material, figure S2a). However, these species were not included in the phylogenetic ANOVA owing to the paucity of phylogenetic information.

In Dasyuromorphia, while the results of the ANOVA and phylogenetic ANOVA were not significant, the average tad/trd scores tended to increase from HC, C, O to I species (electronic supplementary material, figure S2d). Notably, the HC marsupial *Thylacinus* presented larger distal molars than those of other dasyuromorphians (electronic supplementary material, figure S2a). However, carnivorous species did not necessarily present larger M_2_/M_1_ scores within Dasyuridae (electronic supplementary material, figure S2c). As several authors have suggested that the marsupial M_2_ is homologous to the placental M_1_ [44], we also compared M_3_/M_2_ among dietary categories to further examine the relevance of the IC model in marsupials (electronic supplementary material, figure S2e and table S5). Carnivorous species tended to have larger distal molars than omnivorous and insectivorous species (electronic supplementary material, figure S2e).

These results indicated that Carnivora, Creodonta and Dasyuromorphia have different patterns of dietary adaptation with respect to molar proportions, but not to carnassial shape. In Creodonta and Dasyuromorphia, the relative molar sizes based on M_2_/M_1_ scores increased (i.e. increasing distal molar: carnassial) as diet became more carnivorous, in contrast with the pattern observed in Carnivora. However, the carnassial shape based on tad/trd decreased as the diet became carnivorous in all three orders. Therefore, in Carnivora, M_2_/M_1_, M_3_/M_1_ and tad/trd were positively correlated and variation in the ratio of carnassial-trigonid size/total molar row (i.e. the relative shearing region) grew larger (figure 2). Therefore, the relative shearing region varies less in Creodonta than in Carnivora (figure 2).

### (c) *Usag-1*- and *Bmp7*-deficient mice

To determine the cause of the unique IC pattern and the strong correlations between M_2_/M_1_ and tad/trd among carnivorans, we investigated the morphological integration or pleiotropic effects [1,2] of the signalling molecules USAG-1 and BMP7, which are hypothetical components of the IC model [13,45]. According to the GLM results, BMP7 affected both M_2_/M_1_ and tad/trd (*p* < 0.05), but not M_3_/M_1_ (figure 3; electronic supplementary material, tables S6–S7). USAG-1 affected both M_2_/M_1_ and M_3_/M_1_ (*p* < 0.05), but not tad/trd. Statistical interactions between the two molecules were not significant. Therefore, BMP7 results in morphological integration or has pleiotropic effects on the two traits: M_2_/M_1_ and tad/trd (figure 3). As *Bmp7* hetero-deficient mice exhibit larger M_2_/M_1_ and tad/trd, it is likely that the pleiotropic effects of BMP7 generated a positive correlation between the parameters. In addition, changes in only M_2_/M_1_ (and not in M_3_/M_1_) induced by BMP7 result in higher variability in the relative M_1_ size or a smaller slope in the IC regression.

### (d) Molecular evolution of BMP7 among Carnivora and dental evolution in the ursid lineage

**Figure 4.**
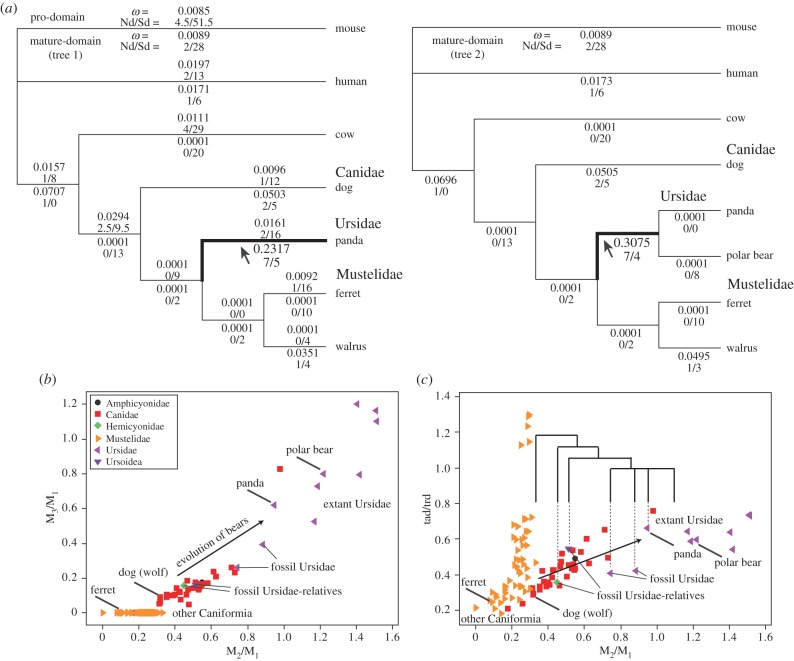
Evolution of BMP7 and dental evolution in ursids. (*a*) The non-synonymous to synonymous rate ratio (*ω*) for each branch, calculated based on the free-ratio model. The estimated numbers of non-synonymous substitutions and synonymous substitutions (Nd/Sd) are also shown based on the ancestral nucleotide sequences inferred using the Bayesian method. The pro-domain and the mature-domain were analysed separately. For the mature-domain, we calculated Nd and Sd using two different trees. In both trees, the mature-domain of BMP7 evolved under relaxed purifying selection only in the ancestral ursid branch (shown as boldface; pointed to with an arrow). (*b*) Plots of M_2_/M_1_ versus M_3_/M_1_ for ursids and other Caniformia. (*c*) Plots of M_2_/M_1_ versus tad/trd for these taxa. Species used for the molecular evolutionary analysis and fossil species important for ursid evolution are marked. Evolution of dentition in ursids is depicted from the lower left to the upper right areas in these plots, similar to the *Bmp7*-hetero-deficient mice. Fossil Ursidae-relatives and fossil Ursidae plotted between dog/ferret and panda, indicating that ursid dentition evolved gradually from the ancestral pattern of caniforms to that of present ursids associated with the evolution of BMP7.

To further understand how *Bmp7* evolved in carnivorans, the numbers and rates of non-synonymous and synonymous substitutions were analysed separately in the pro-domain and the mature-domain. We determined that the mature-domain of BMP7 evolved under significantly higher *ω* ratios in the ancestral ursid branch than other branches, while the pro-domain in this branch evolved under strong purifying selection (small *ω* ratios), similar to other branches (figure 4*a*; electronic supplementary material, tables S8–S9). It should be noted that while non-synonymous mutations were significantly more frequent than in the other branches, the *ω* ratio was still less than 1 in the ursid branch.

The direction of dental evolution from the mustelid–ursid common ancestor to Ursidae is represented in figure 4*b*,*c*. Ferrets, dogs and other caniforms formed a group in the morphospace, while ursids occupied a distinct region (figure 4*b*,*c*). A species of canid (*Otocyon megalotis*) which possesses four lower molars was plotted closer to ursid. In addition, fossil Ursidae relatives (i.e. members of Hemicyonidae and Ursoidea, which are outgroups of extant Ursidae) were closer to caniforms, and fossil ursids were grouped between extant Ursidae and outgroups (figure 4*b*,*c*). These results indicate that M_2_/M_1_ and tad/trd scores increased during ursid evolution.

## 4. Discussion

### (a) Pattern of dietary adaptation of the molars of three mammalian orders

The relative molar sizes (M_2_/M_1_) and carnassial shape (tad/trd) exhibited parallel evolution, reflecting dietary evolution in carnivorans, creodonts and dasyuromorphians, indicating that these traits are important for dietary adaptations (ANOVA with Tukey's test and phylogenetic ANOVA: figure 1; electronic supplementary material, figures S1–S2 and table S4). We observed two opposing patterns of adaptation: carnivorans tended to exhibit increases in mesial molars during carnivorous adaptation, but creodonts and dasyuromorphians tended to exhibit increased distal molars (i.e. they presented enlarged carnassials for a carnivorous diet; figure 1*a*,*b*). The difference in the observed evolutionary patterns in relative molar sizes can be explained by which molar was the carnassial in the ancestor (i.e. the mesial molar in Carnivora and distal molar in Creodonta and Dasyuromorphia). However, the pattern of adaptation in carnassial shape was similar among these three orders (figure 1*a*,*b*). The relative proportions of shearing and grinding regions have been previously investigated in Carnivora by measuring the total molar row [23–25], but these dental measurements are a combination of two dental traits: relative molar size (e.g. M_2_/M_1_) and carnassial shape (tad/trd).

### (b) Variability in relative molar sizes in Carnivora

For relative molar sizes, the carnivoran IC pattern is distinct from those of other mammals with smaller slopes for the M_2_/M_1_ versus M_3_/M_1_ regression (figure 1*a*; electronic supplementary material, table S2) [13–20]. In the Carnivora, many extant taxa lost their M_3_, except for Canidae and Ursidae. Focusing on these carnivoran taxa, which have three lower molars, the relationship between M_2_/M_1_ and M_3_/M_1_ was observed to be unique among mammals. That is, the slopes in carnivoran taxa (Canidae: 0.45; Canidae + Ursidae: 0.90; Ursidae: 1.08) were lower than the observed range of interspecific variation at the family or higher taxonomic level for other mammals (1.17–3.269) [13,18–20] (electronic supplementary material, table S2). According to this variability, the relative M_1_ (carnassial) size, which is important for the relative shearing and grinding surfaces, can vary more than in other mammals (figure 1).

### (c) Correlation between two traits important for dietary adaptation in Carnivora

Furthermore, relative molar sizes (M_2_/M_1_) and carnassial shape (tad/trd) were extensively correlated in carnivorans but not in creodonts or dasyuromorphians (figure 1*b*,*c*; electronic supplementary material, table S3). A positive correlation between M_2_/M_1_ and tad/trd facilitates carnivoran dietary adaptations in both carnivorous and omnivorous directions because the relative shearing and grinding functions change. During carnivorous adaptation, the relative carnassial size (relative M_1_ size in relation to total molar row) and relative trigonid size (shearing region) in the carnassial correspondingly increase. During omnivorous adaptation, the relative size of the distal molars and talonid of the first molar (all of which have grinding functions) can correspondingly increase. Conversely, a negative correlation between these two traits, if present, can facilitate dietary adaptation in Creodonta and Dasyuromorphia. This is because the carnassial is located mesially in Carnivora but distally in Creodonta and Dasyuromorphia. A correlation between M_2_/M_1_ and tad/trd was only observed in Carnivora and may be a characteristic of the group (electronic supplementary material, table S3 and text S5). This correlation may facilitate the parallel evolution of dentition during dietary adaptation in many carnivoran families (electronic supplementary material, table S4). However, in Creodonta and Dasyuromorphia, these two traits were not highly correlated. According to the unique IC pattern (smaller slope) and the correlation between relative molar size and carnassial shape, carnivoran dentition can vary greatly in the relative proportion of shearing and grinding regions (figure 2). For example, among families that present three molars, the relative shearing region ranged from 0.3 to 0.7 in Canidae (even excluding *Otocyon megalotis*, which has a score of 0.17, but four molars) and from 0.2 to 0.45 in Hyaenodontidae. As a result, all families except for Mephitidae include more than one dietary category (electronic supplementary material, table S4 and text S6).

We did not observe a correlation between M_2_/M_1_ and tad/trd in Creodonta and Dasyuromorphia. However, when *Sarcophilus* was excluded from the Dasyuromorphia dataset, we observed a significant correlation (slope < 0 in contrast with carnivorans; electronic supplementary material, figure S2). Therefore, relative molar sizes and molar shape also evolved simultaneously in Dasyuromorphia, while the relative molar size pattern was opposite to that of Carnivora. However, these correlations were relatively weaker (as they were heavily influenced by one species) than those of carnivorans. This was also true when we used M_3_/M_2_ scores for the molar proportion in Dasyuromorphia (electronic supplementary material, table S5; slope = −1.208, *r* = −0.429, *p* = 0.06). These results suggest that the two traits are not highly correlated, and the pattern of adaptation observed in Creodonta and Dasyuromorphia was not easily facilitated by existing patterns of variation. Consequently, the relative shearing region varied less in Creodonta and Dasyuromorphia than in Carnivora (figure 2).

### (d) Molar morphology of BMP7-hetero-deficient mice

The results obtained using genetically modified mice suggest that the patterns of integration and variability caused by BMP7 are consistent with the evolutionary pattern of carnivoran dentition that influences M_2_/M_1_ and tad/trd, but not M_3_/M_1_ (figures 1 and 3; electronic supplementary material, tables S2–S3; for USAG-1, see electronic supplementary material, text S7). If this molecule evolves in carnivorans, then the proportion of shearing and grinding regions should vary greatly (figure 2). However, a positive correlation between M_2_/M_1_ and tad/trd mediated by BMP7 inhibits non-carnivoran patterns of dietary adaptation, such as that of creodonts. This is because creodonts exhibit the opposite adaptation pattern; their adaptation is facilitated by a negative correlation between M_2_/M_1_ and tad/trd (figure 1*b*,*c*). This integration mechanism can promote a positive correlation between M_2_/M_1_ and tad/trd in Carnivora, and inhibit a negative correlation between the parameters in Creodonta (figure 1*b*; electronic supplementary material, table S3). This integration may be caused by the same series of molecules involved in inhibition/activation signalling pathways during the formation of both primary and secondary enamel knots (i.e. the same series of molecules affects both teeth and cusp formation [13,29,46]). Accordingly, the relative sizes of the mesial cusps in molars and mesial molars within molar rows can be correlated. Therefore, carnivorans have an advantage over other mammals because they may easily evolve various diets. In particular, their dental function may evolve rapidly because the location of the carnassial in the molar row fits the developmental mechanism in mammals.

### (e) Molecular evolution of BMP7 and morphological evolution of molars in the ursid lineage, with implications for the cause of the carnivoran inhibitory cascade pattern

Our molecular evolutionary analysis revealed that only the mature-domain of the BMP7 evolved under a high *ω* ratio along the ursid lineage, although the ratio was still less than one. Two hypotheses can explain this result: a reduction in the functional importance of the BMP7 (i.e. relaxed purifying selection) and positive selection at the particular time of the branch. Because the pro-domain of the BMP7 evolved under strict purifying selection in the ursid lineage and the *Bmp7* knockout is lethal in mice [47,48], a reduction in the functional importance of BMP7 is unlikely, and therefore the positive selection hypothesis is favoured.

It has been hypothesized that inhibitory molecules have various diffusibilities and the evolution of one of them generates a unique IC pattern in canids [15]. The pro-domain of BMP7 interacts with the extracellular matrix and thereby inhibits its diffusion, and the mature-domain functions as a signalling molecule [40–42]. Therefore, the molecular evolutionary results indicate that the strength of signalling changed over time, but diffusibility did not change in the ancestral ursid lineages. This conclusion is consistent with the observation that *Bmp7*-hetero-deficient mice, which may present low signalling ability, exhibited higher M_2_/M_1_ and tad/trd scores, and hence displayed a trend similar to that of the evolution of ursids. In the original experiment by Kavanagh *et al*., on which the IC model is based, all inhibitory molecules were simultaneously blocked during mouse development, or Activin A and BMP4 were added *in vitro* [13]. However, we speculate that if another inhibitory molecule with low diffusibility (or its antagonistic activation molecules) is increased or decreased, or if its affinity for its antagonist changes, the dental pattern would be different from the original model (slope = 2).

Morphological data from fossils and extant carnivorans are consistent with this scenario. The two morphological traits, M_2_/M_1_ and tad/trd, which are affected by BMP7, changed gradually from extant carnivorous non-ursid caniformia, fossil ursid-relatives and extant ursids (figure 4*b*,*c*). Ursids have been considered the exception of the IC model because they exhibit M_1_ < M_2_ > M_3_ [14]. However, our results indicate that ursids evolved gradually from other carnivorans along their unique IC pattern, presenting larger talonids and distal molars (with a particular increase in M_2_ size), similar to the BMP7-hetero-deficient mice (figure 3). Therefore, we conclude that the evolution of enigmatic ursid dentition [14] was caused, or at least influenced, by positive selection of the mature-domain of BMP7.

This evolutionary pattern is similar to the Canidae–Ursidae variability (the small slope of the M_2_/M_1_ versus M_3_/M_1_ regression and significant correlation between M_2_/M_1_ and tad/trd; figure 1). Therefore, we hypothesized that the unique carnivoran molar variability and IC pattern (small slope of the M_2_/M_1_ versus M_3_/M_1_ regression; electronic supplementary material, tables S1–S2) results from evolutionary changes in the expression or function of low-diffusible inhibitory molecules or their antagonists, such as BMP7, which affects M_2_/M_1_, but not M_3_/M_1_. BMP7 effects on carnivoran evolution are also evidenced by the covariation between M_2_/M_1_ and tad/trd, which are modified in BMP7-deficient mice (figures 1–4). The high variability in mesial molar sizes ([15] and this study) and that in mesial cusp positions and sizes [49] in carnivorans may be caused by high variability in low-diffusible signalling molecules during inhibition/activation patterning [29].

### (f) Advantage of carnivoran dentition: evolutionary and developmental context

Previous studies have suggested that the evolutionary success of carnivorans is attributable to the evolutionary versatility of their dentition; specifically, the proportion of shearing and grinding functions can be changed [8]. Our findings not only support this hypothesis (figure 2), but also indicate that a developmental mechanism enhances the effect of the versatility. That is, a change in only one signalling molecule can generate phenotypic evolutions that correspondingly contribute to dietary adaptation. In addition, variability in these traits is consistent with the functional changes required for various dietary adaptations. This results from the fact that the M_1_ of carnivoran ancestors evolved into the carnassial. Consequently, adaptations to different dietary habits and the parallel evolution of dental proportions are more frequently observed in carnivorans than in creodonts and dasyuromorphians (electronic supplementary material, figures S1–S2 and table S4). In other words, carnivoran dietary adaptations can occur more easily and rapidly. We speculate that this is related to their developmental mechanism, such as the positive correlation between M_2_/M_1_ and tad/trd, which facilitates carnivoran dental evolution. When a new niche emerges, this developmental mechanism enables taxa to occupy the new niche faster than their competitors can, improving competition. According to a previous study, an increase in diversity or the extinction of higher taxa depends on the rate of species origination relative to the background extinction rate [50]. Accordingly, the fact that dietary adaptations and dental evolution are facilitated by their underlying developmental mechanism may have been a critical factor in the success of carnivorans.

Our results show that the adaptation pattern and developmental mechanisms of carnivorans give them advantages over creodonts with respect to dietary adaptation. The extinction of creodonts and the high current carnivoran diversity may be caused, or at least influenced, by the adaptation pattern and developmental mechanism discussed above. In addition, we suggested a mechanism to explain the enigmatic dental pattern of ursids [14]. These results were generated by a combination of morphological comparisons of museum specimens, the use of a genetically modified model organism and a molecular evolutionary analysis. This approach could serve as an example for elucidating the developmental and genetic basis of unique morphological characters of non-model organisms and fossil species.

## Supplementary Material

Supplementary materials

## Data accessibility

Data and material availability: most relevant data are within the paper and in the electronic supplementary material. The nucleotide sequences of Bmp7 genes of the examined species were retrieved from public databases (for accession numbers, see electronic supplementary material, text S4).
