# Supplementary material for: Unique pattern of dietary adaptation in the dentition of Carnivora: its advantage and developmental origin
Source: Proc Biol Sci. 2016 Jun 15;283(1832):20160375. doi: 10.1098/rspb.2016.0375 (PMC4920314; doi:10.1098/rspb.2016.0375)
Supplement: Supplementary materials [file rspb20160375supp1.pdf]

Supplementary Text

Text S1: Specimens examined

We used mandible specimens including living and fossil species deposited in the National Museum of Nature and Science (Japan), American Museum of Natural History, the United States National Museum of Natural History, University of California Museum of Paleontology, and the Museum of Vertebrate Zoology at the University of California at Berkeley. Because we focused on molar and cusp sizes, we used specimens in which all lower molars and their carnassials were not worn out.

Text S2: Reference list for information for diet

Dietary information (electronic supplementary material, table S1) is cited from the literatures [52–77]. While dietary information on fossil species was based on their morphology, including dental morphology, a thorough discussion of other characters such as heeled talonids (talonids that acquired shearing function) and OPC (oriented patch count), or other cranial morphology are presented. Therefore, dietary information is considered to be fundamentally independent from our morphological indices such as M_2/M_1 and tad/trd.

Text S3: Reference list for information for phylogenetic relationships

Phylogenetic trees used for phylogenetic Anova (electronic supplementary material, figures S4,6) are reconstructed from literatures listed below: family-level phylogeny for the entire order of Carnivora was carried out as per the information provided in [78]; members within the Canidae family [79–81]; Dasyuromorphia [82]; Eupleridae [83–85]; Herpestidae [83,86]; Hyaenidae [83]; Mephitidae [83,87]; Mustelidae [83, 88,89], Procyonidae [83,90,91]; Ursidae [92]; Viverridae [83]; Hyaenodontidae [93–97], Oxyaenidae [98,99]. A phylogenetic tree of the Figure 3c is based on previous reports [100–102].

Text S4: Molecular evolution of BMP7

The nucleotide sequences of *Bmp7* genes were retrieved from public databases with the following accession numbers; human *Homo sapiens*: AK291186 (Genbank), mouse *Mus musculus*: BC10771 (Genbank), dog *Canis lupus*: NM_001197052 (Genbank), cow *Bos taurus*: NM_001206015 (Genbank), walrus *Odobenus rosmarus*: XM_004416538 (Genbank), panda *Ailuropoda melanoleuca*: XM_002915544 (Genbank), ferret *Mustela putorius*: ENSMPUT00000006533 (Ensembl http://www.ensembl.org/). Recently, a polar bear genome assembly (UrsMar1.0) was released and RefSeq annotation of the

polar bear BMP7 sequence is available under the following accession no., XM_008699503.1. However, the pro-domain of the RefSeq-annotated polar bear BMP7 sequence differs greatly from that of other animals analysed in this study, indicating erroneous annotation. Therefore, we downloaded the UrsMar1.0 genome assembly data and searched the BMP7 sequence with BLASTN program ver. 2.2.29 [103] using panda, walrus and dog BMP7 sequences as queries. The BMP7 gene consists of 7 exons [104], and exons 2–7 were found on scaffold 148 of the UrsMar1.0 genome assembly. However, we could not find the exon 1, which may have been due to incompleteness of the UrsMar1.0 genome assembly. For these reasons, we analysed only mature-domain of the polar bear BMP7 sequence. These BMP7 sequences were aligned using L-INS-i program in the MAFFT package ver. 6.240 [105,106] with manual adjustments, and then divided into two domains following the procedure of [107]. The CODEML program in the PAML4.4 package [108] was used to calculate synonymous and nonsynonymous substitutions based on widely accepted phylogenetic trees (Figs. 3a and S3). Several models listed below were compared: (i) all branches had evolved under different ω ratios (free-ratio model), (ii) all branches had evolved under the same ω ratio (one-ratio model), or (iii) all branches except for the ursid branch had evolved under the same ω ratio, but the ursid branch had evolved under different selective pressure

(two-ratio model). In all models, the transition/transversion rates were not fixed, and the F3×4 model was used for codon usage biases in which nucleotide frequencies at three codon positions are used to calculate codon frequencies. Likelihood ratio tests were performed to compare between one-ratio models and two-ratio models, and the significance of differences was evaluated by calculating twice the log-likelihood difference assuming that it follows a χ^2 distribution, with the number of degrees of freedom equal to the difference in the numbers of free parameters between models. The numbers of nonsynonymous substitutions(N_d) and synonymous substitutions (S_d) were calculated by the method of Nei and Gojobori [109] based on the ancestral nucleotide sequences inferred by the Bayesian method [110]. The test of homogeneity of nonsynonymous/synonymous substitution ratios [111] was also applied to examine whether selective pressure on the ursid branch can be considered homogeneous in comparison with that on other carnivore branches.

Text S5: Correlation between M_2/M_1 and tad/trd is a characteristic of Carnivora

However, only the Carnivora exhibited such a correlation within all families except for Mephitidae and Procyonidae, which exhibit mainly omnivorous pattern (no highly carnivorous species) (electronic supplementary material, table S3). The absence of a

significant correlation in Mephitidae and Procyonidae may have resulted from these dietary variations noted above. Therefore, the correlation between M_2/M_1 and tad/trd is considered to be characteristic of Carnivora

Text S6: Canidae evolvability

Among all families examined, Canidae exhibits the strongest correlations between M_2/M_1 and tad/trd (electronic supplementary material, table S3). In addition, the effect of diet on M_2/M_1 and tad/trd in Canidae is the most extensive among all families; that is, all dietary pairs were significant except for between HC and C (electronic supplementary material, table S4). According to this developmental constraint that facilitates adaptation pattern, Canidae has particularly high evolvability in dental function adapting to diet.

TextS7: phenotype of USAG-1 hetero-deficient mice

USAG-1 hetero-deficient mice show larger mesial molar (Figure 2). Interestingly, that USAG-1 (ectodin) is an inhibitor that induces a reduction in the size of distal molars. In other words, the decrease in the expression of inhibitors seems to generate a phenotype opposite to that expected based on the IC model. It is also interesting to note that

USAG-1 and BMP7 deficient mice show phenotypes opposite to those of the corresponding hetero-deficient mice [39]. Thus there may be a more complex phenomenon, including molecular interactions between inhibitors and activators, such as a reaction-diffusion system.

References

52. Gaubert P. Nandiniidae. In: Wilson DE, Mittermeier RA, editors. Handbook of the mammals of the world 1. Carnivores. Barcelona: Lynx Edicions; 2009. pp. 50–53.

53. Sunquist ME, Sunquist FC. In: Wilson DE, Mittermeier RA, editors. Handbook of the mammals of the world 1. Carnivores. Barcelona: Lynx Edicions; 2009. pp. 54–169.

54. Gaubert P. In: Wilson DE, Mittermeier RA, editors. Handbook of the mammals of the world 1. Carnivores. Barcelona: Lynx Edicions; 2009. pp. 170–173.

55. Jennings AP, Veron G. In: Wilson DE, Mittermeier RA, editors. Handbook of the mammals of the world 1. Carnivores. Barcelona: Lynx Edicions; 2009. pp. 174–233.

56. Holekamp KE, Kolowski JM. In: Wilson DE, Mittermeier RA, editors. Handbook of the mammals of the world 1. Carnivores. Barcelona: Lynx Edicions; 2009. pp. 234–261.

57. Gilchrist JS, Jennings AP, Veron G, Gavallini P. In: Wilson DE, Mittermeier RA, editors. Handbook of the mammals of the world 1. Carnivores. Barcelona: Lynx Edicions; 2009. pp. 262–329.

58. Goodman SM. In: Wilson DE, Mittermeier RA, editors. Handbook of the mammals of the world 1. Carnivores. Barcelona: Lynx Edicions; 2009. pp. 330–351.

59. Sillero-Zubiri C. In: Wilson DE, Mittermeier RA, editors. Handbook of the mammals of the world 1. Carnivores. Barcelona: Lynx Edicions; 2009. pp. 352–447 .

60. Garshelis DL. In: Wilson DE, Mittermeier RA, editors. Handbook of the mammals of the world 1. Carnivores. Barcelona: Lynx Edicions; 2009. pp. 448–497.

61. Wei F, Zhang Z. In: Wilson DE, Mittermeier RA, editors. Handbook of the mammals of the world 1. Carnivores. Barcelona: Lynx Edicions; 2009. pp. 498–503.

62. Kays R. In: Wilson DE, Mittermeier RA, editors. Handbook of the mammals of the world 1. Carnivores. Barcelona: Lynx Edicions; 2009. pp. 504–531.

63. Dragoo JW. In: Wilson DE, Mittermeier RA, editors. Handbook of the mammals of the world 1. Carnivores. Barcelona: Lynx Edicions; 2009. pp. 532–563.

64. Larivière S, Jennings AP. In: Wilson DE, Mittermeier RA, editors. Handbook of the mammals of the world 1. Carnivores. Barcelona: Lynx Edicions; 2009. pp. 564–658.

65. Flores DA, Giannini N, Abdala F. 2006 Comparative postnatal ontogeny of the skull in the Australidelphian Metatherian *Dasyurus albopunctatus* (Marsupialia: Dasyuromorphia: Dasyuridae). *J. Morph.* **267**, 426–440.

66. Wroe S, McHenry C, Thomason J. 2005 Bite club: comparative bite force in big biting mammals and the prediction of predatory behaviour in fossil taxa. *Proc. R Soc. B.* **272**, 619–625.

67. Wroe S, Milne N. 2007 Convergence and remarkably consistent constraint in the evolution of carnivore skull shape. *Evolution* **61**, 1251–1260.

68. Taylor R. 1986 Notes on the diet of the carnivorous mammals of the upper Henty river region, western Tasmania. *Papers Proc. R Soc. Tasmania* **120**, 7–10.

69. Kitchener DJ. 1981 Breeding, diet and habitat preference of *Phascogale calura* (Gould, 1844) (Marsupialia: Dasyuridae) in the southern wheat belt, western Australia. *Rec. West Aust. Mus.* **9**, 173–186.

70. Grossek GL, Woolley PA, Menzies JI. 2010 The diet of some New Guinean dasyurid marsupials. *Aust. Mammal.* **32**, 145–155.

71. van Deusen HM. 2005 Feeding habits of *Planigale* (Marsupialia, Dasyuridae). *J. Mammal.* **50**, 616–618.

72. Gilfillan SL. 2001 An ecological study of a population of *Psendantechinus macdonnellensis* (Marsupialia: Dasyuridae) in central Australia. I. Invertebrate food supply, diet and reproductive strategy. *Wildlife Res.* **28**, 469–480.

73. Morlo M, Gunnell F, Nagel D. Ecomorphological analysis of carnivoran guilds. In: Goswami A, Friscia A, editors. Carnivoran evolution. Cambridge: Cambridge University Press; 2010; pp. 269–311.

74. Chester SGB, Bloch JI, Secord R, Boyer DM. 2010 A new small-bodied species of

Palaeonictis (Creodonta, Oxyaenidae) from the Paleocene-Eocene thermal maximum. *J. Mammal Evol.* **17**, 227–243.

75. Attard MRG, Chamoli U, Ferrara TL, Rogers TL, Wroe S. 2011 Skull mechanics and implications for feeding behaviour in a large marsupial carnivore guild: the thylacine, Tasmanian devil and spotted-tailed quoll. *J. Zool.* **285**, 292–300.

76. Oldfield CC, McHenry CR, Clausen PD, Chamoli U, Parr WCH, Stynder DD, et al. 2012 Finite element analysis of ursid cranial mechanics and the predation of feeding behaviour in the extinct giant *Agriotherium africanum*. *J. Zool.* **286**, 163–170.

77. Solé F, Gheerbrant E, Godinot M. 2011 New data on the Oxyaenidae from the Early Eocene of Europe; biostratigraphic, paleobiogeographic and paleoecoogic implications. *Paleontol. Electronica.* **14**, 1–41.

78. Flynn JJ, Finarelli JA, Zehr S, Hsu J, Nedbal MA. 2005 Molecular phylogeny of the Carnivora (Mammalia): assessing the impact of increased sampling on resolving enigmatic relationsips. *Syst. Biol.* **54**, 317–337.

79. Bardeleben C, Moore RL, Wayne RK. 2005 A molecular phylogeny of the Canidae based on six nuclear loci. *Mol. Phyl. Evol.* **37**, 815–831.

80. Zrzavý J, ŘIčánková V. 2004 Phylogeny of recent Canidae (Mammalia, Carnivora): relative reliability and utility of morphological and molecular datasets. *Zool. Scr.* **33**,

311–333.

81. Prevosti FJ. 2011 Phylogeny of the large extinct South American Canids (Mammalia, Carnivora, Canidae) using a “total evidence” approach. **Cladistics** *26*, 456–481.

82. Cardillo M, Bininda-Emonds ORP, Boakes E, Purvis A. 2004 A species-level phylogenetic supertree of marsupials. *J. Zool.* **264**, 11–31.

83. Agnarsson I, Kuntner M, May-Collado L. 2010 Dogs, cats and kin: a molecular species-level phylogeny of Carnivora. *Mol. Phyl. Evol.* **54**, 726–745.

84. Yoder AD, Burns MM, Zehr S, Delefosse T, Veron G, et al. 2003 Single origin of Malagasy Carnivora from an African ancestor. *Nature* **421**, 734–737.

85. Gaubert P, Wozencraft WC, Cordeiro-Estrela P, Veron G. 2005 Mosaics of convergences and noise in morphological phylogenies: what’s in a viverrid-like carnivorans? *Syst. Biol.* **54**, 865–894.

86. Patou ML, Mclenachan PA, Morley CG, Couloux A, Jennings AP, Veron G. 2009 Molecular phylogeny of the Herpestidae (Mammalia, Carnivora) with a special emphasis of the Asian Herpestes. *Mol. Phyl. Evol.* **53**, 69–80.

87. Finarelli JA. 2008 A total evidence phylogeny of the Arctoidea (Carnivora: Mammalia): relationships among basal taxa. *J. Mammal. Evol.* **15**, 231–259.

88. Sato JJ, Wolsan M, Minami S, Hosoda T, Sinaga MH, Hiyama K, et al. 2009 Deciphering and dating the red panda’s ancestry and early adaptive radiation of Musteloidea. *Mol. Phyl. Evol.* **53**, 907–922.

89. Sato JJ, Wolsan M, Prevosti FJ, D'Elía G, Begg C, Begg K, et al. 2012 Evolutionary and biogeographic history of weasel-like carnivorans (Musteloidea). *Mol. Phyl. Evol.* **63**, 745–757.

90. Koepfli KP, Gompper ME, Eizirik E, Ho CC, Linden L, Maldonado JE, et al. 2007 Phylogeny of the Procyonidae (Mammalia: Carnivora): molecules, morphology and the great American interchange. *Mol. Phyl. Evol.* **43**, 1076–1095.

91. Helgen KM, Kays R, Helgen LE, Tsuchiya-Jerep MT, Pinto CM, Koepfli KP, et al. 2009 Taxonomic boundaries and geographic distributions revealed by an integrative systematic overview of the mountain coatis, *Nasuella* (Carnivora: Procyonidae). *Small Carnivore Conser.* **41**, 65–74.

92. Pagès M, Calvignac S, Klein C, Paris M, Hughes S, Hänni C. 2008 Combined analysis of fourteen nuclear genes refines the Ursidae phylogeny. *Mol. Phyl. Evol.* **47**, 73–83.

93. Gunnell GF. Creodonta. In: Janis CM, Scott KM, Jacobs LL, editors. Evolution of tertiary mammals of North America volume1: terrestrial carnivores, ungulates, and

ungulatelike mammals. Cambridge: Cambridge University Press; pp. 91–109; 1998.

94. Solé F. 2013 New proviverrine genus from the Early Eocene of Europe and the first phylogeny of Late Palaeocene-Middle Eocene hyaenodontidans (Mammalia). *J. Syst. Paleontol.* **11**, 375–398.

95. Egi N, Holroyd PA, Tsubamoto T, Soe AN, Takai M, Ciochon RL. 2005 Proviverrine hyaenodontids (Creodonta: Mammalia) from the Eocene of Myanmar and a phylogenetic analysis of the proviverrines from the Para-Tethys area. *J. Syst. Paleontol.* **3**, 337–358.

96. Peigné S, Morlo M, Chaimanee Y, Ducrocq S, Tun ST, Jaeger JJ. 2007 New discoveries of hyaenodontids (Creodonta, Mammalia) from the Pondaung formation, middle Eocene, Myanmar– paleobiogeographic implications. *Geodiversitas* **29**, 441–458.

97. Solé F, Lhuillier J, Adaci M, Bensalah M, Mahboubi MH, Tabuce R. 2014 The hyaenodontidans from the Gour Lazib area (?Early Eocene, Algeria): implications concerning the systematics and the origin of the Hyainailourinae and Teratodontinae. *J. Syst. Paleontol.* **12**, 303–322.

98. Stefen C. 1997 The enamel of Creodonta, Arctocyonidae, and Mesonychidae (Mammalia), with special reference to the appearance of Hunter-Schreger-Bands.

Paläontologische Zeitschrift **71**, 291–303.

99. Solé F, Gheerbrant E, Godinot M. 2011 New data on Oxyaenidae from the Early Eocene of Europe; biostratigraphic, paleobiogeographic and paleoecologic implications. *Palaeontologia Electoronica* **14**, 13A.

100. Hunt RM. Ursidae. In: Janis CM, Scott KM, Jacobs LL, editors. Evolution of Tertiary mammals of North America. Cambridge: Cambridge University Press; 1998; pp. 174–195.

101. Finarelli JA. 2008 A total evidence phylogeny of the Arctoidea (Carnivora: Mammalia): relationships among basal taxa. *J. Mammal. Evol.* **15**, 231–259.

102. Abella J, Alba DM, Robles JM, Valenciano A, Rotgers C, Carmona R, et al. 2012 Kretzoiarctos gen. nov., the oldest member of the giant panda clade. *Plos One* **7**, e48985.

103. Camacho C, Coulouris G, Avagyan V, Ma N, Papadopoulos J, Bealer K, et al. 2009 BLAST+: architecture and applications. *BMC Bioinfor.* **10**, 421.

104. Ozkaynak E, Schnegelsberg PN, Jin DF, Clifford GM, Warren FD, Drier EA, et al. 1992 Osteogenic protain-2: a new member for the transforming growth factor-β superfamily expressed early in embryogenesis. *J. Biol. Chem.* **267**, 25220–7.

105. Katoh K, Misawa K, Kuma K, Miyama T. 2002 MAFFT: a novel method for rapid

multiple sequence alignment based on fast Fourier transform. *Nucleic Acids Res.* **30**, 3059–3066.

106. Katoh K, Kuma K, Toh H, Miyata T. 2005 MAFFT version 5: improvement in accuracy of multiple sequence alignment. *Nucleic Acids Res.* **33**, 511–518.

107. Nematollahi L, Mahboudi F, Rahimpour A, Jahandar H, Khalaj V. 2013 A novel human bone morphogenetic protein-7 variant with an enriched heparin-binding site. *Mol. Biol.* **47**, 399–405.

108. Yang Z. 2007 PAML 4: phylogenetic analysis by maximum likelihood. *Mol. Biol. Evol.* **24**, 1586–1591.

109. Nei M, Gojobori T. 1986 Simple methods for estimating the numbers of synonymous and nonsynonymous nucleotide substitutions. *Mol. Biol. Evol.* **3**, 418–426.

110. Yang Z, Kumar S, Nei M. 1995 A new method of inference of ancestral nucleotide and amino acid sequences. *Genetics* **141**, 1641–1650.

111. Kishida T, Thewissen JGM. 2012 Evolutionary changes of the importance of olfaction in cetaceans based on the olfactory marker protein gene. *Gene* **492**, 349–353.

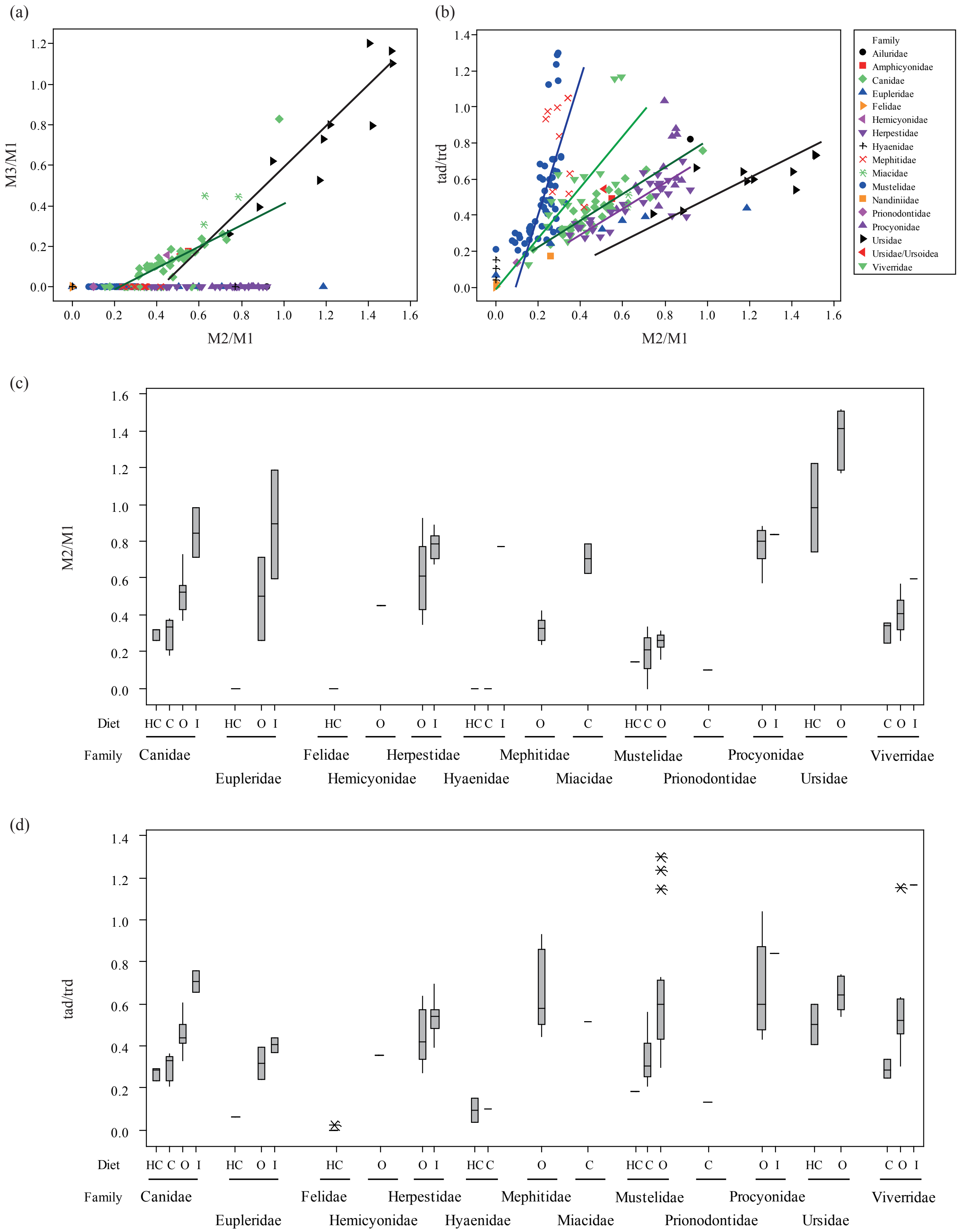

Supplementary Figure S1

Variation in relative molar sizes and M1 shape within carnivoran families.
(a) Plots of M2/M1 versus M3/M1 color-coded by families and RMA regressions within Ursidae and Canidae (excluding Otocyon).
(b) Plots of the M2/M1 versus tad/trd color-coded by families and RMA regressions within Mustelidae, Viverridae, Canidae, Herpestidae, and Ursidae. (c) Box plots of M2/M1 for each dietary category within each family. For all box-plots in the present study, boxes indicate quartiles, central-lateral bars indicate averages, vertical bars indicate the range of the specimens, and asterisks indicate outliers. Within all families, carnivorous species tend to have lower M2/M1 scores, i.e., larger M1, and omnivorous and insectivorous species tend to have larger M2/M1 scores, i.e., more equal-sized molars. (d) Box plots of tad/trd for each dietary category within each family. Within all families, carnivorous species tend to have lower tad/trd scores, i.e., larger trigonid and smaller talonid of M1, and omnivorous and insectivorous species tend to have larger tad/trd scores, i.e., smaller trigonid and larger talonid of M1.

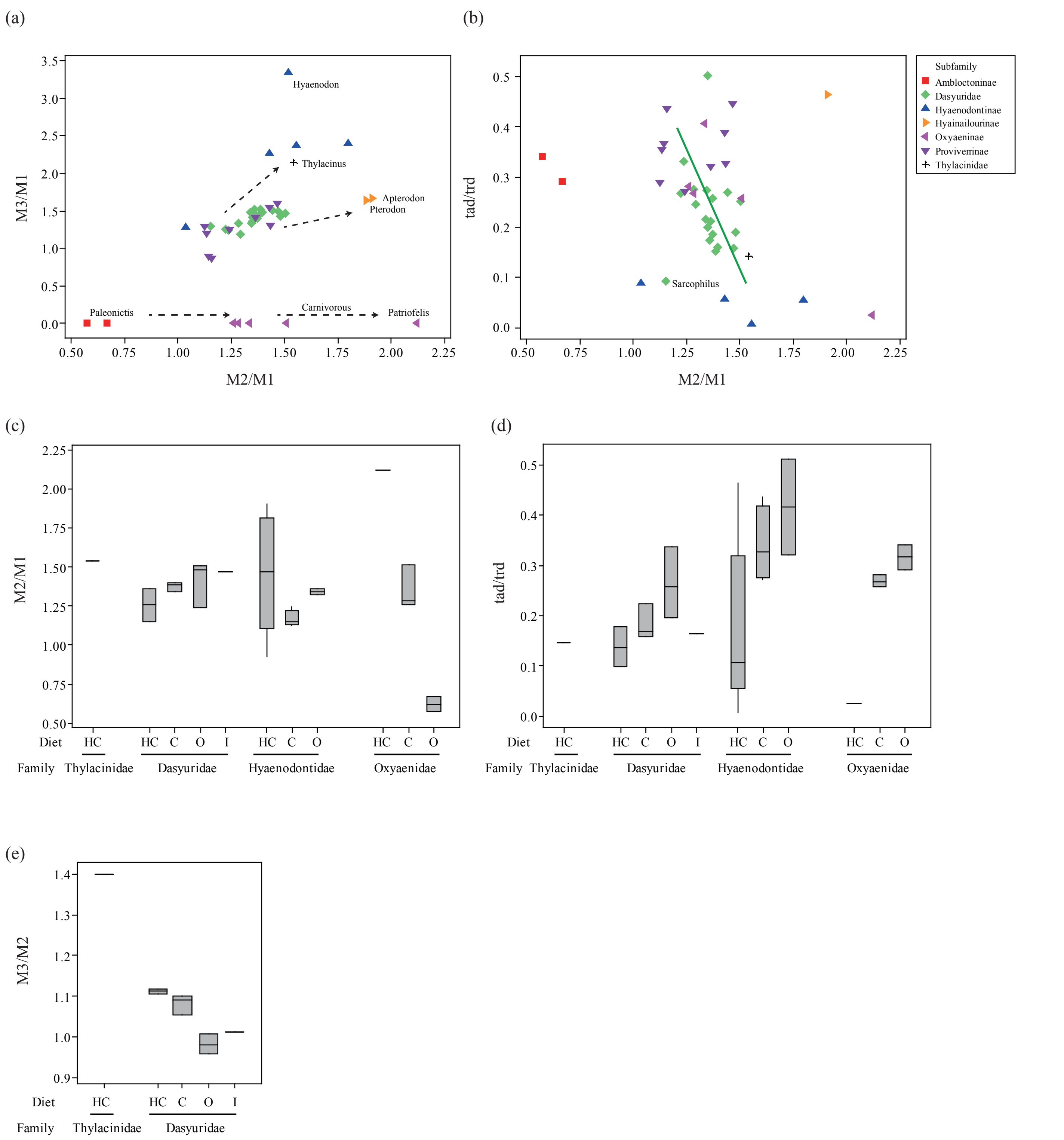

Supplementary Figure S2

Variation in relative molar sizes and M1 shape within creodont and dasyuromorphian families.
(a)Plots of M2/M1 versus M3/M1 color-coded by families. (b) Plots of the M2/M1 versus tad/trd color-coded by families with RMA regressions within Dasyuromorphia (excluding Sarcophilus). Dotted arrowed lines indicate several evolutionary cases: from omnivorous-carnivorous Proviverrinae hyaenodonts to more carnivorous Hyainailourinae hyaenodonts; from omnivorous Ambloctoninae oxyaenids, carnivorous Oxyaeninae species, to hyper-carnivorous Patriofelis; from other dasyuromorphian species to hyper-carnivorous Thylacinus. (c) Box plots of M2/M1 for each dietary category within each family. Carnivorous species tend to have larger distal molars in Oxyaenidae, but the pattern is not clear the other families. (d) Box plots of tad/trd for each dietary category within each family. Within all families, carnivorous species tend to have lower tad/trd scores, i.e., larger trigonid and smaller talonid of M1, and omnivorous and insectivorous species tend to have larger tad/trd scores, i.e., smaller trigonid and larger talonid of M1. (e) Box plots of M3/M2 for each dietary category within each dasyuromorphian family. Enlargement of distal molars in carnivorous species is cleariy seen by comparison of M2/M1 scores.

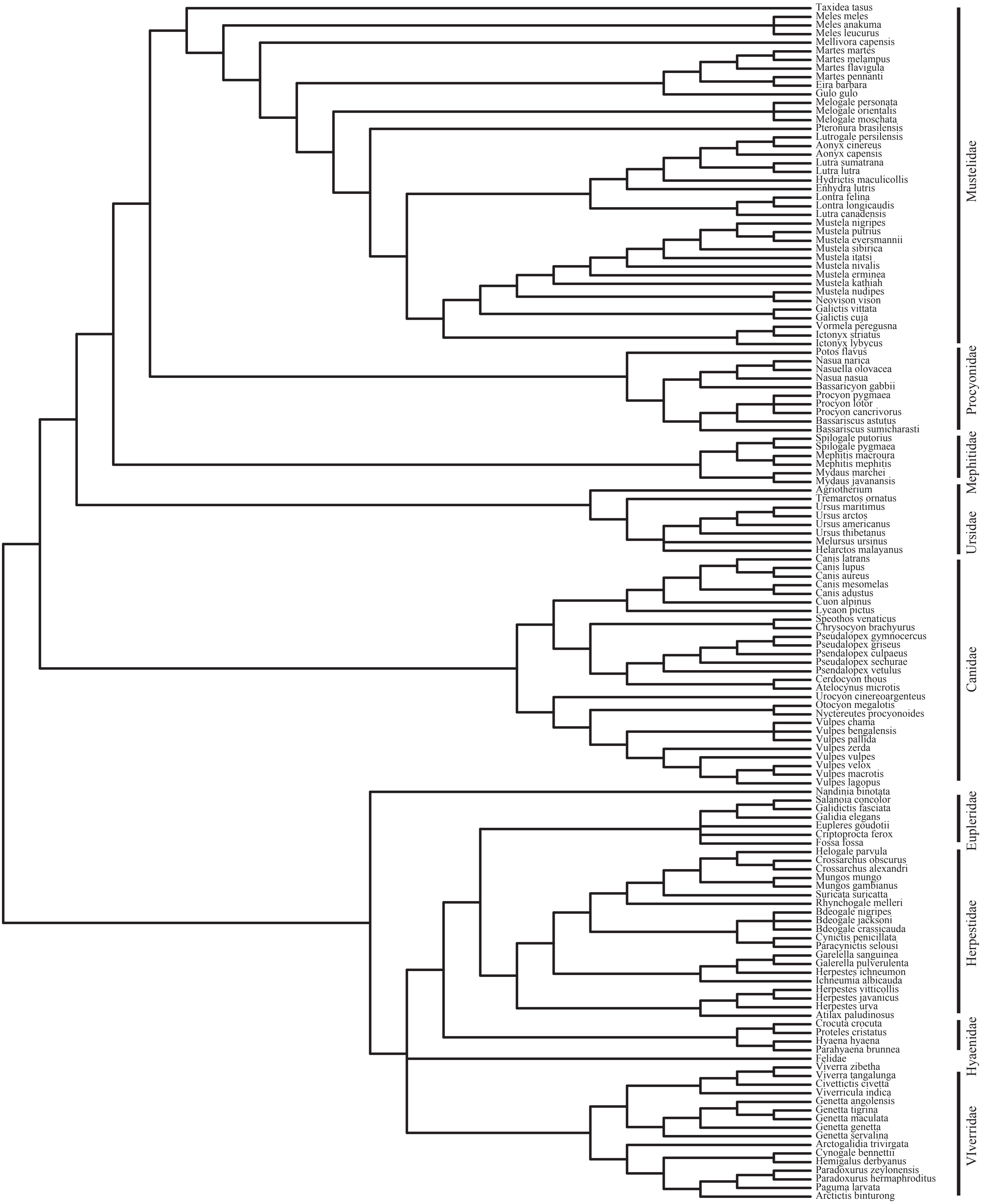

Supplementary Figure S3
Phylogenetic tree of the Carnivora used for the phylogenetic ANOVA.

(a)

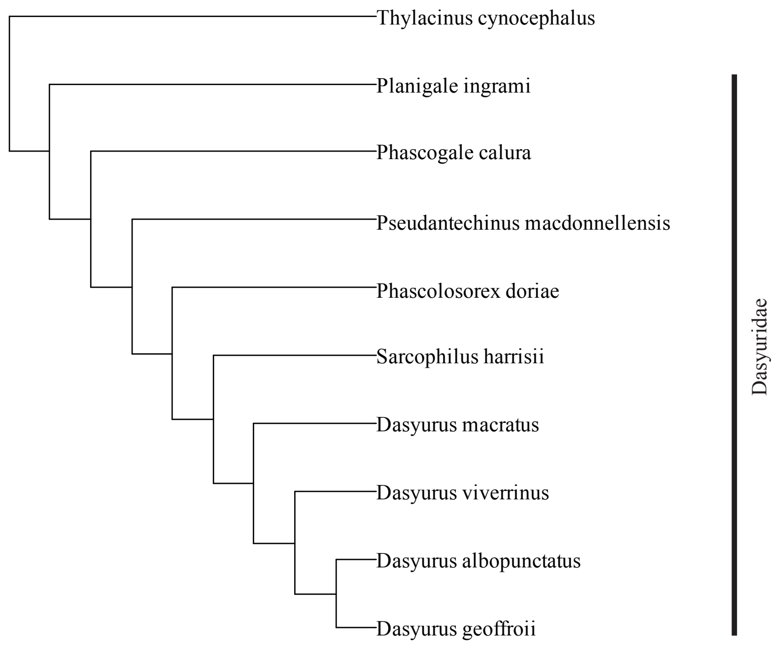

(b)

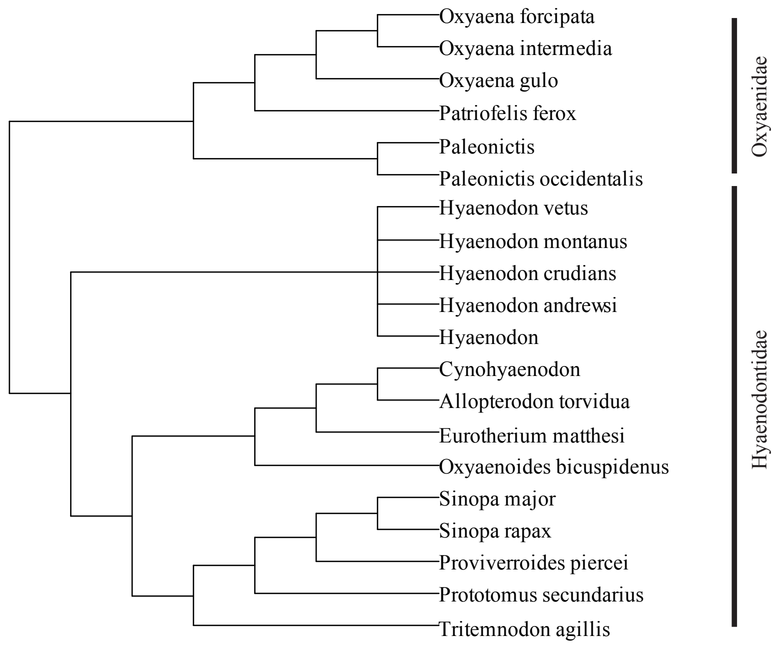

Supplementary Figure S4

Phylogenetic trees used for the phylogenetic ANOVA.

(a) Tree of the Dasyuromorphia. (b) Tree of the Creodonta.

Supplementary Table S1. Species examined in this study, and data used. Diet categories are according to cited literatures shown.

Number	Species	Family	Subspecies etc.	Order	M2/M1	M2/M1SD	M3/M1	M3/M1SD	Tad/Trd	Tad/TrdSD	Trd/total molar row	N	Diet	Reference
1	*Ailurus fulgens*	Ailuridae	Ailuridae	Carnivora	0.919	0.018	0.000	0.000	0.818	0.049	0.287	2	He	61
2	†*Daphoenus*	Amphicyonidae	Amphicyonidae	Carnivora	0.547		0.176		0.491		0.389	1		
3	*Atelocynus microtis*	Canidae	Canidae	Carnivora	0.545	0.044	0.157	0.043	0.427	0.008	0.412	3	O	59
4	*Canis adustus*	Canidae	Canidae	Carnivora	0.513	0.128	0.173	0.058	0.452	0.036	0.409	4	O	59
5	*Canis aureus*	Canidae	Canidae	Carnivora	0.427	0.049	0.115	0.023	0.424	0.022	0.455	11	O	59
6	*Canis latrans*	Canidae	Canidae	Carnivora	0.376	0.033	0.099	0.022	0.359	0.019	0.499	52	C	59
7	*Canis lupus*	Canidae	Canidae	Carnivora	0.319	0.031	0.091	0.015	0.287	0.010	0.551	28	HC	59
8	*Canis mesomelas*	Canidae	Canidae	Carnivora	0.367	0.028	0.100	0.020	0.419	0.033	0.480	20	O	59
9	*Canis rufus*	Canidae	Canidae	Carnivora	0.355		0.088		0.316		0.527	1		
10	*Canis simensis*	Canidae	Canidae	Carnivora	0.429		0.076		0.426		0.466	1		
11	*Cerdocyon thos*	Canidae	Canidae	Carnivora	0.550	0.063	0.166	0.032	0.434	0.028	0.407	5	O	59
12	*Chrysocyon brachyurus*	Canidae	Canidae	Carnivora	0.467	0.012	0.184	0.014	0.444	0.055	0.419	3	O	59
13	*Cuon alpinus*	Canidae	Canidae	Carnivora	0.260	0.000	0.000	0.000	0.236	0.003	0.643	2	HC	59
14	*Hesperocyon*	Canidae	Canidae	Carnivora	0.406		0.139		0.340		0.483	1		
15	*Hesperocyon gregarius*	Canidae	Canidae	Carnivora	0.462		0.102		0.338		0.478	1		
16	*Lycalopex culpaeus*	Canidae	Canidae	Carnivora	0.419	0.036	0.120	0.028	0.382	0.008	0.470	10	O	59
17	*Lycalopex griseus*	Canidae	Canidae	Carnivora	0.501	0.052	0.139	0.024	0.390	0.003	0.439	15	O	59
18	*Lycalopex gymnocercus*	Canidae	Canidae	Carnivora	0.531	0.029	0.131	0.020	0.432	0.000	0.420	9	O	59
19	*Lycalopex sechurae*	Canidae	Canidae	Carnivora	0.537	0.046	0.166	0.018	0.457	0.006	0.403	5	O	59
20	*Lycalopex vetulus*	Canidae	Canidae	Carnivora	0.712	0.128	0.257	0.116	0.652	0.026	0.307	6	I	59
21	*Lycaon pictus*	Canidae	Canidae	Carnivora	0.319	0.022	0.062	0.029	0.293	0.052	0.560	7	HC	59
22	*Nyctereutes procyonoides*	Canidae	Canidae	Carnivora	0.473	0.036	0.046	0.043	0.518	0.049	0.434	44	O	59
23	*Otocyon megalotis*	Canidae	Canidae	Carnivora	0.979	0.057	0.828	0.065	0.759	0.092	0.166	7	I	59
24	*Speothos venaticus*	Canidae	Canidae	Carnivora	0.177	0.049	0.000	0.000	0.209	0.032	0.703	4	C	59
25	*Urocyon cinereoargenteus*	Canidae	Canidae	Carnivora	0.533	0.040	0.147	0.035	0.537	0.042	0.387	32	O	59
26	*Urocyon littoralis*	Canidae	Canidae	Carnivora	0.470	0.026	0.132	0.016	0.473	0.037	0.424	3		
27	*Vulpes bengalensis*	Canidae	Canidae	Carnivora	0.612	0.033	0.235	0.045	0.601	0.033	0.338	2	O	59
28	*Vulpes chama*	Canidae	Canidae	Carnivora	0.627		0.208		0.464		0.372	1	O	59
29	*Vulpes lagopus*	Canidae	Canidae	Carnivora	0.313	0.042	0.050	0.042	0.323	0.018	0.554	31	C	59
30	*Vulpes macrotis*	Canidae	Canidae	Carnivora	0.397	0.058	0.104	0.009	0.354	0.011	0.492	4	O	59
31	*Vulpes pallida*	Canidae	Canidae	Carnivora	0.729		0.232		0.496		0.341	1	O	59
32	*Vulpes velox*	Canidae	Canidae	Carnivora	0.391	0.017	0.099	0.017	0.333	0.018	0.504	7	O	59
33	*Vulpes vulpes*	Canidae	Canidae	Carnivora	0.351	0.019	0.101	0.010	0.333	0.001	0.517	3	C	59
34	*Vulpes zerda*	Canidae	Canidae	Carnivora	0.580	0.050	0.168	0.035	0.528	0.037	0.375	6	O	59
35	*Antechinus flavipes*	Dasyuridae	Dasyuridae	Dasyuromorphia	1.373	0.044	1.419	0.006	0.257	0.001	0.129	2		
36	*Antechinus godmani*	Dasyuridae	Dasyuridae	Dasyuromorphia	1.373	0.034	1.401	0.094	0.187	0.034	0.121	2		
37	*Antechinus hageni*	Dasyuridae	Dasyuridae	Dasyuromorphia	1.346	0.100	1.336	0.150	0.273	0.001	0.129	2		
38	*Antechinus leo*	Dasyuridae	Dasyuridae	Dasyuromorphia	1.363	0.042	1.439	0.067	0.212	0.010	0.134	2		
39	*Dasyuroides byrnei*	Dasyuridae	Dasyuridae	Dasyuromorphia	1.442	0.038	1.505	0.066	0.268	0.023	0.184	3		
40	*Dasyurus albopunctatus*	Dasyuridae	Dasyuridae	Dasyuromorphia	1.396	0.093	1.473	0.128	0.159	0.013	0.155	4	C	65
41	*Dasyurus geoffroii*	Dasyuridae	Dasyuridae	Dasyuromorphia	1.388	0.069	1.512	0.069	0.153	0.030	0.180	3	C	66,67
42	*Dasyurus hallucatus*	Dasyuridae	Dasyuridae	Dasyuromorphia	1.349	0.028	1.360	0.048	0.199	0.030	0.150	4		
43	*Dasyurus maclatus*	Dasyuridae	Dasyuridae	Dasyuromorphia	1.357	0.071	1.520	0.147	0.173	0.006	0.183	2	HC	66,67
44	*Dasyurus viverrinus*	Dasyuridae	Dasyuridae	Dasyuromorphia	1.341	0.049	1.477	0.079	0.216	0.044	0.171	4	C	66,68
45	*Murexia longicaudata*	Dasyuridae	Dasyuridae	Dasyuromorphia	1.282	0.037	1.333	0.072	0.275	0.021	0.137	2		
46	*Myoictis melas*	Dasyuridae	Dasyuridae	Dasyuromorphia	1.294	0.086	1.183	0.033	0.245	0.008	0.137	2		
47	*Neophascogale lorentzii*	Dasyuridae	Dasyuridae	Dasyuromorphia	1.347		1.404		0.501		0.136	1		
48	*Phascogale calura*	Dasyuridae	Dasyuridae	Dasyuromorphia	1.505		1.457		0.252		0.145	1	O	69
49	*Phascoloorex doriae*	Dasyuridae	Dasyuridae	Dasyuromorphia	1.239		1.242		0.330		0.149	1	O	70
50	*Planigale ingrami*	Dasyuridae	Dasyuridae	Dasyuromorphia	1.482	0.093	1.424	0.180	0.189		0.161	2	O	71
51	*Pseudantechinus macdonnellensis*	Dasyuridae	Dasyuridae	Dasyuromorphia	1.470	0.079	1.492	0.126	0.157	0.042	0.145	2	I	72
52	*Sarcophilus harrisii*	Dasyuridae	Dasyuridae	Dasyuromorphia	1.151	0.013	1.292	0.039	0.092	0.037	0.228	4	HC	66,68
53	*Sminthopsis virginiae*	Dasyuridae	Dasyuridae	Dasyuromorphia	1.224	0.038	1.250	0.025	0.268	0.036	0.124	2		
54	*Cryptoprocta ferox*	Eupleridae	Eupleridae	Carnivora	0.000		0.000		0.063		0.941	1	HC	58
55	*Eupleres goudotii*	Eupleridae	Eupleridae	Carnivora	1.188		0.000		0.440		0.317	1	I	58
56	*Fossa fossa*	Eupleridae	Eupleridae	Carnivora	0.708	0.083	0.000	0.000	0.392	0.048	0.421	3	O	58
57	*Galidia elegans*	Eupleridae	Eupleridae	Carnivora	0.260		0.000		0.240		0.640	1	O	58
58	*Galidictis fasciata*	Eupleridae	Eupleridae	Carnivora	0.503	0.051	0.000	0.000	0.320	0.010	0.504	2	O	58
59	*Salanoia concolor*	Eupleridae	Eupleridae	Carnivora	0.596		0.000		0.371		0.457	1	I	58
60	*Acinonyx jubatus*	Felidae	Felidae	Carnivora	0.000		0.000		0.022		0.979	1	HC	73
61	*Caracal caracal*	Felidae	Felidae	Carnivora	0.000		0.000		0.000		1.000	1	HC	73
62	*Catopuma badia*	Felidae	Felidae	Carnivora	0.000		0.000		0.000		1.000	1	HC	53
63	*Catopuma temminckii*	Felidae	Felidae	Carnivora	0.000		0.000		0.000		1.000	1	HC	53
64	*Felis chaus*	Felidae	Felidae	Carnivora	0.000		0.000		0.000		1.000	1	HC	53
65	*Felis margarita*	Felidae	Felidae	Carnivora	0.000		0.000		0.000		1.000	1	HC	53
66	*Felis nigripes*	Felidae	Felidae	Carnivora	0.000		0.000		0.000		1.000	1	HC	53
67	*Felis silvestris*	Felidae	Felidae	Carnivora	0.000		0.000		0.000		1.000	1	HC	53
68	*Herpailurus yaguarondi*	Felidae	Felidae	Carnivora	0.000		0.000		0.000		1.000	1	HC	73
69	*Leopardus pardalis*	Felidae	Felidae	Carnivora	0.000		0.000		0.000		1.000	1	HC	73
70	*Leopardus tigrinus*	Felidae	Felidae	Carnivora	0.000		0.000		0.000		1.000	1	HC	73
71	*Leopardus wiedii*	Felidae	Felidae	Carnivora	0.000		0.000		0.000		1.000	1	HC	73
72	*Leptailurus serval*	Felidae	Felidae	Carnivora	0.000		0.000		0.000		1.000	1	HC	53
73	*Lynx canadensis*	Felidae	Felidae	Carnivora	0.000		0.000		0.000		1.000	1	HC	53
74	*Lynx pardinus*	Felidae	Felidae	Carnivora	0.000		0.000		0.000		1.000	1	HC	53
75	*Lynx rufus*	Felidae	Felidae	Carnivora	0.000		0.000		0.000		1.000	1	HC	53
76	*Oncifelis colocolo*	Felidae	Felidae	Carnivora	0.000		0.000		0.000		1.000	1		
77	*Oncifelis geoffroyi*	Felidae	Felidae	Carnivora	0.000		0.000		0.000		1.000	1		
78	*Otocolobus manul*	Felidae	Felidae	Carnivora	0.000		0.000		0.000		1.000	1	HC	53
79	*Panthera leo*	Felidae	Felidae	Carnivora	0.000		0.000		0.000		1.000	1	HC	73
80	*Panthera onca*	Felidae	Felidae	Carnivora	0.000		0.000		0.000		1.000	1	HC	73
81	*Panthera pardus*	Felidae	Felidae	Carnivora	0.000		0.000		0.000		1.000	1	HC	73
82	*Panthera tigris*	Felidae	Felidae	Carnivora	0.000		0.000		0.000		1.000	1	HC	
83	*Pardofelis marmorata*	Felidae	Felidae	Carnivora	0.000		0.000		0.000		1.000	1	HC	53
84	*Prionailurus bengalensis*	Felidae	Felidae	Carnivora	0.000		0.000		0.000		1.000	1	HC	53
85	*Prionailurus planiceps*	Felidae	Felidae	Carnivora	0.000		0.000		0.000		1.000	1		
86	*Prionailurus viverrinus*	Felidae	Felidae	Carnivora	0.000		0.000		0.000		1.000	1		
87	*Profelis aurata*	Felidae	Felidae	Carnivora	0.000		0.000		0.000		1.000	1	HC	53
88	*Puma concolor*	Felidae	Felidae	Carnivora	0.000		0.000		0.000		1.000	1	HC	73
89	*Uncia uncia*	Felidae	Felidae	Carnivora	0.000		0.000		0.000		1.000	1		
90	†*Cephalogale minor*	Hemicyonidae	Hemicyonidae	Carnivora	0.449		0.157		0.354		0.460	1	O	
91	*Atilax paludinosus*	Herpestidae	Herpestinae	Carnivora	0.544	0.031	0.000	0.000	0.418	0.047	0.457	3	O	57
92	*Bdeogale crassicauda*	Herpestidae	Herpestinae	Carnivora	0.817	0.053	0.000	0.000	0.520	0.086	0.362	2	I	57
93	*Bdeogale jacksoni*	Herpestidae	Herpestinae	Carnivora	0.763		0.000		0.623		0.349	1	O	57
94	*Bdeogale nigripes*	Herpestidae	Herpestinae	Carnivora	0.920	0.073	0.000	0.000	0.537	0.042	0.339	2	O	57
95	*Crossarchus alexandri*	Herpestidae	Mungotinae	Carnivora	0.817	0.026	0.000	0.000	0.590	0.053	0.346	2	O	57
96	*Crossarchus obscurus*	Herpestidae	Mungotinae	Carnivora	0.763	0.052	0.000	0.000	0.565	0.040	0.362	2	O	57
97	*Cynictis penicillata*	Herpestidae	Herpestinae	Carnivora	0.698	0.010	0.000	0.000	0.471	0.039	0.400	2	I	57
98	*Paracynictis selousi*	Herpestidae	Herpestinae	Carnivora	0.835	0.064	0.000	0.000	0.396	0.023	0.390	2	I	57
99	*Dologale dybowskii*	Herpestidae	Mungotinae	Carnivora	0.773	0.069	0.000	0.000	0.578	0.014	0.357	2	O	57
100	*Galerella pulverulenta*	Herpestidae	Herpestinae	Carnivora	0.350		0.000		0.286		0.576	1	O	57

Supplementary Table S1. Species examined in this study, and data used. Diet categories are according to cited literatures shown (continued).

Number	Species	Family	Subspecies etc.	Order	M2/M1	M2/M1SD	M3/M1	M3/M1SD	Tad/Trd	Tad/TrdSD	Trd/total molar row	N	Diet	Reference
101	*Galerella sanguinea*	Herpestidae	Herpestinae	Carnivora	0.400		0.000		0.332		0.536	1	O	57
102	*Helogale hirtula*	Herpestidae	Mungotinae	Carnivora	0.755	0.016	0.000	0.000	0.508	0.064	0.378	2		
103	*Helogale parvula*	Herpestidae	Mungotinae	Carnivora	0.675		0.000		0.532		0.390	1	I	57,73
104	*Herpestes brachyurus*	Herpestidae	Herpestinae	Carnivora	0.613	0.003	0.000	0.000	0.424	0.004	0.435	2	O	57
105	*Herpestes edwardsii*	Herpestidae	Herpestinae	Carnivora	0.495		0.000		0.277		0.524	1	O	57
106	*Herpestes fuscus*	Herpestidae	Herpestinae	Carnivora	0.458	0.038	0.000	0.000	0.315	0.011	0.521	2		
107	*Herpestes ichneumon*	Herpestidae	Herpestinae	Carnivora	0.420	0.032	0.000	0.000	0.270	0.026	0.555	3	O	57
108	*Herpestes javanicus*	Herpestidae	Herpestinae	Carnivora	0.377	0.036	0.000	0.000	0.355	0.008	0.536	3	O	57
109	*Herpestes naso*	Herpestidae	Herpestinae	Carnivora	0.537	0.077	0.000	0.000	0.303	0.040	0.499	2		
110	*Herpestes pulverulentus*	Herpestidae	Herpestinae	Carnivora	0.443		0.000		0.353		0.512	1		
111	*Herpestes semitorguatus*	Herpestidae	Herpestinae	Carnivora	0.389		0.000		0.329		0.542	1		
112	*Herpestes urva*	Herpestidae	Herpestinae	Carnivora	0.450	0.067	0.000	0.000	0.373	0.061	0.502	3	O	57
113	*Herpestes vitticollis*	Herpestidae	Herpestinae	Carnivora	0.602	0.104	0.000	0.000	0.457	0.045	0.428	2	O	
114	*Ichneumia albicauda*	Herpestidae	Herpestinae	Carnivora	0.790	0.064	0.000	0.000	0.550	0.068	0.360	3	I	57,73
115	*Mungos gambianus*	Herpestidae	Mungotinae	Carnivora	0.699		0.000		0.635		0.360	1	O	57
116	*Mungos mungo*	Herpestidae	Mungotinae	Carnivora	0.777	0.062	0.000	0.000	0.546	0.046	0.364	4	I	57,73
117	*Rhynchogale melleri*	Herpestidae	Herpestinae	Carnivora	0.883		0.000		0.696		0.313	1	I	57
118	*Suricata suricatta*	Herpestidae	Mungotinae	Carnivora	0.729	0.058	0.000	0.000	0.575		0.367	2	I	57
119	*Crocuta crocuta*	Hyaenidae	Hyaenidae	Carnivora	0.000	0.000	0.000	0.000	0.039	0.026	0.962	2	HC	73
120	*Hyaena hyaena*	Hyaenidae	Hyaenidae	Carnivora	0.000	0.000	0.000	0.000	0.152	0.011	0.868	2	HC	73
121	*Parahyaena brunnea*	Hyaenidae	Hyaenidae	Carnivora	0.000	0.000	0.000	0.000	0.103	0.045	0.906	2	C	56
122	*Proteles cristata*	Hyaenidae	Hyaenidae	Carnivora	0.772	0.321	0.000	0.000				3	I	56
123	†*Allopterodon torvidus*	Hyaenodontidae	Proviverrinae	Creodonta	1.241		1.248		0.270		0.282	1	C	73
124	†*Apterodon*	Hyaenodontidae	Hyainailourinae	Creodonta	1.913		1.666		0.464		0.248	1	HC	77
125	†*Cynohyaenodon*	Hyaenodontidae	Proviverrinae	Creodonta	1.125	0.100	1.295	0.182	0.288	0.054	0.294	2	C	
126	†*Eurotherium matthesi*	Hyaenodontidae	Proviverrinae	Creodonta	1.133		1.200		0.354		0.266	1	HC	73
127	†*Hyaenodon*	Hyaenodontidae	Hyaenodontinae	Creodonta	1.518		3.338					1	HC	
128	†*Hyaenodon andrewsi*	Hyaenodontidae	Hyaenodontinae	Creodonta	1.797		2.390		0.054		0.437	1	HC	
129	†*Hyaenodon crudians*	Hyaenodontidae	Hyaenodontinae	Creodonta	1.037		1.284		0.088		0.355	1	HC	
130	†*Hyaenodon montanus*	Hyaenodontidae	Hyaenodontinae	Creodonta	1.427		2.263		0.057		0.457	1	HC	
131	†*Hyaenodon vetus*	Hyaenodontidae	Hyaenodontinae	Creodonta	1.556	0.328	2.370	0.723	0.007	0.010	0.478	2	HC	
132	†*Masrasector aegypticum*	Hyaenodontidae	Proviverrinae	Creodonta	1.436		1.300		0.327		0.262	1		
133	†*Oxyaenoides bicuspidenus*	Hyaenodontidae		Creodonta	0.931		0.000		0.125			1	HC	73
134	†*Prototomus secundarius*	Hyaenodontidae	Proviverrinae	Creodonta	1.362		1.406		0.320		0.283	1	O	
135	†*Proviverroides piercei*	Hyaenodontidae		Creodonta	1.319		1.131		0.511		0.217	1	O	
136	†*Pterodon africanus*	Hyaenodontidae	Hyainailourinae	Creodonta	1.883		1.639					1	HC	77
137	†*Quercitherium*	Hyaenodontidae		Creodonta	1.085		1.202		0.235		0.296	1		
138	†*Sinopa hians*	Hyaenodontidae	Proviverrinae	Creodonta	1.429		1.544		0.388		0.280	1		
139	†*Sinopa major*	Hyaenodontidae	Proviverrinae	Creodonta	1.156		0.869		0.435		0.200	1	C	73
140	†*Sinopa rapax*	Hyaenodontidae	Proviverrinae	Creodonta	1.145	0.055	0.895	0.174	0.366	0.018	0.216	3	C	73
141	†*Sinopa strenua*	Hyaenodontidae	Proviverrinae	Creodonta	1.468		1.595		0.445		0.272	1		
142	†*Tritemnodon agilis*	Hyaenodontidae		Creodonta	1.225	0.019	1.283	0.109	0.213	0.050	0.302	2	HC	73
143	*Conepatus chinga*	Mephitidae	Mephitidae	Carnivora	0.337		0.000		1.048		0.365	1		
144	*Conepatus humboldtii*	Mephitidae	Mephitidae	Carnivora	0.244		0.000		0.974		0.407	1		
145	*Conepatus leuconotus*	Mephitidae	Mephitidae	Carnivora	0.338		0.000		1.047		0.365	1		
146	*Conepatus mesoleucus*	Mephitidae	Mephitidae	Carnivora	0.288		0.000		0.996		0.389	1		
147	*Mephitis macroura*	Mephitidae	Mephitidae	Carnivora	0.342		0.000		0.519		0.490	1	O	63
148	*Mephitis mephitis*	Mephitidae	Mephitidae	Carnivora	0.351	0.054	0.000	0.000	0.630	0.054	0.454	5	O	63
149	*Mydaus javanensis*	Mephitidae	Mephitidae	Carnivora	0.300	0.060	0.000	0.000	0.835	0.189	0.419	2	O	63
150	*Mydaus marchei*	Mephitidae	Mephitidae	Carnivora	0.237		0.000		0.930		0.419	1	O	63
151	*Spilogale putorius*	Mephitidae	Mephitidae	Carnivora	0.416		0.000		0.442		0.490	1	O	63
152	*Spilogale pygmaea*	Mephitidae	Mephitidae	Carnivora	0.268		0.000		0.529		0.516	1	O	63
153	†*Miacis latidens*	Miacidae	Miacidae	Carnivora	0.620	0.032	0.306	0.024	0.451	0.111	0.358	2		
154	†*Vulpavus australis*	Miacidae	Miacidae	Carnivora	0.626		0.448		0.514		0.318	1	C	
155	†*Vulpavus palustris*	Miacidae	Miacidae	Carnivora	0.783		0.444					1	C	73
156	*Aonyx cinereus*	Mustelidae	Lutrinae	Carnivora	0.307	0.039	0.000	0.000	0.722	0.007	0.444	2	O	64
157	*Aonyx capensis*	Mustelidae	Lutrinae	Carnivora	0.238		0.000		0.696		0.492	1	O	64
158	*Arctonyx albogularis*	Mustelidae	Melinae	Carnivora	0.289		0.000		1.289		0.461	1		
159	*Arctonyx collaris*	Mustelidae	Melinae	Carnivora	0.251	0.029	0.000	0.000	1.126	0.102	0.339	2		
160	*Eira barbara*	Mustelidae	Martinae	Carnivora	0.299	0.068	0.000	0.000	0.318	0.059	0.376	2	O	64
161	*Enhydra lutris*	Mustelidae	Lutrinae	Carnivora	0.308	0.026	0.000	0.000	0.720	0.032	0.584	2	O	64
162	*Galictis cuja*	Mustelidae	Galictidinae	Carnivora	0.201	0.083	0.000	0.000	0.268	0.029	0.445	2	C	64,73
163	*Galictis vittata*	Mustelidae	Galictidinae	Carnivora	0.217	0.039	0.000	0.000	0.336	0.057	0.657	2	C	64
164	*Gulo gulo*	Mustelidae	Martinae	Carnivora	0.141	0.010	0.000	0.000	0.183	0.034	0.615	3	HC	
165	*Hydrictis maculicollis*	Mustelidae	Lutrinae	Carnivora	0.257		0.000		0.563		0.741	1	O	64
166	*Ictonyc libycus*	Mustelidae	Galictidinae	Carnivora	0.273	0.029	0.000	0.000	0.304	0.055	0.509	3	C	64
167	*Ictonyx striatus*	Mustelidae	Galictidinae	Carnivora	0.253	0.002	0.000	0.000	0.331	0.052	0.603	2	O	64
168	*Lontra canadensis*	Mustelidae	Lutrinae	Carnivora	0.292		0.000		0.642		0.478	1	O	64
169	*Lontra felina*	Mustelidae	Lutrinae	Carnivora	0.229		0.000		0.534		0.551	1	O	64
170	*Lontra longicaudis*	Mustelidae	Lutrinae	Carnivora	0.259		0.000		0.486		0.487	1	O	64
171	*Lutra lutra*	Mustelidae	Lutrinae	Carnivora	0.275	0.013	0.000	0.000	0.711	0.038	0.459	2	O	64
172	*Lutra macullicolis*	Mustelidae	Lutrinae	Carnivora	0.252		0.000		0.595		0.501	1		
173	*Lutra sumatrana*	Mustelidae	Lutrinae	Carnivora	0.223		0.000		0.595		0.513	1	O	64
174	*Lutrogale perspicillata*	Mustelidae	Lutrinae	Carnivora	0.269		0.000		0.605		0.491	1	O	64
175	*Martes americana*	Mustelidae	Martinae	Carnivora	0.228	0.011	0.000	0.000	0.444	0.022	0.564	4		
176	*Martes flavigula*	Mustelidae	Martinae	Carnivora	0.245	0.040	0.000	0.000	0.333	0.060	0.603	3	C	64
177	*Martes foina*	Mustelidae	Martinae	Carnivora	0.235	0.049	0.000	0.000	0.419	0.002	0.571	3	C	64
178	*Martes martes*	Mustelidae	Martinae	Carnivora	0.279	0.019	0.000	0.000	0.435	0.025	0.545	2	C	64
179	*Martes melampus*	Mustelidae	Martinae	Carnivora	0.265		0.000		0.459		0.542	1	C	64
180	*Martes pennanti*	Mustelidae	Martinae	Carnivora	0.283	0.054	0.000	0.000	0.382	0.021	0.564	2	C	64
181	*Martes zibellina*	Mustelidae	Martinae	Carnivora	0.293	0.046	0.000	0.000	0.555	0.125	0.512	2	C	64
182	*Meles anakuma*	Mustelidae	Melinae	Carnivora	0.293		0.000		1.146		0.360	1	O	64
183	*Meles leucurus*	Mustelidae	Melinae	Carnivora	0.292		0.000		1.298		0.337	1	O	64
184	*Meles meles*	Mustelidae	Melinae	Carnivora	0.286	0.045	0.000	0.000	1.235	0.174	0.348	3	O	64
185	*Mellivora capensis*	Mustelidae	Mellivorinae	Carnivora	0.000		0.000		0.211		0.826	1	C	64,73
186	*Melogale moschata*	Mustelidae	Helictidinae	Carnivora	0.210	0.047	0.000	0.000	0.609	0.096	0.514	3	O	64
187	*Melogale orientalis*	Mustelidae	Helictidinae	Carnivora	0.211	0.035	0.000	0.000	0.453	0.067	0.568	2	O	64
188	*Melogale personata*	Mustelidae	Helictidinae	Carnivora	0.243	0.014	0.000	0.000	0.408	0.065	0.571	2	O	64
189	*Mustela africana*	Mustelidae	Mustelinae	Carnivora	0.160	0.019	0.000	0.000	0.262	0.006	0.683	2		
190	*Mustela erminea*	Mustelidae	Mustelinae	Carnivora	0.108	0.034	0.000	0.000	0.287	0.018	0.702	3	C	64
191	*Mustela eversmannii*	Mustelidae	Mustelinae	Carnivora	0.104	0.031	0.000	0.000	0.206	0.030	0.751	2	C	64
192	*Mustela felipei*	Mustelidae	Mustelinae	Carnivora	0.185		0.000		0.364		0.619	1		
193	*Mustela frenata*	Mustelidae	Mustelinae	Carnivora	0.092	0.013	0.000	0.000	0.300	0.030	0.704	3	C	64
194	*Mustela itatsi*	Mustelidae	Mustelinae	Carnivora	0.158		0.000		0.327		0.650	1	O	64
195	*Mustela kathiah*	Mustelidae	Mustelinae	Carnivora	0.162	0.017	0.000	0.000	0.297	0.036	0.664	2	O	64
196	*Mustela nigripes*	Mustelidae	Mustelinae	Carnivora	0.077	0.020	0.000	0.000	0.254	0.033	0.740	2	C	64
197	*Mustela nivalis*	Mustelidae	Mustelinae	Carnivora	0.135		0.000		0.240		0.711	1	C	64
198	*Mustela nudipes*	Mustelidae	Mustelinae	Carnivora	0.112		0.000		0.274		0.706	1	C	64
199	*Mustela putorius*	Mustelidae	Mustelinae	Carnivora	0.092		0.000		0.245		0.736	1	C	64
200	*Mustela sibirica*	Mustelidae	Mustelinae	Carnivora	0.158	0.017	0.000	0.000	0.312	0.037	0.658	2	C	64

Supplementary Table S1. Species examined in this study, and data used. Diet categories are according to cited literatures shown (continued).

Number	Species	Family	Subspecies etc.	Order	M2/M1	M2/M1SD	M3/M1	M3/M1SD	Tad/Trd	Tad/TrdSD	Trd/total molar row	N	Diet	Reference
201	*Neovison vison*	Mustelidae	Mustelinae	Carnivora	0.164		0.000		0.334		0.644	1	O	64
202	*Poecilogale albinucha*	Mustelidae	Galictidinae	Carnivora	0.096		0.000					1		
203	*Pteronura brasiliensis*	Mustelidae	Lutrinae	Carnivora	0.241	0.007	0.000	0.000	0.481	0.024	0.544	2	O	64
204	*Taxidea taxus*	Mustelidae	Taxidiinae	Carnivora	0.279	0.052	0.000	0.000	0.562	0.092	0.501	3	C	64
205	*Vormela peregusna*	Mustelidae	Galictidinae	Carnivora	0.239		0.000		0.260		0.641	1	C	64
206	*Nandinia binotata*	Nandiniidae		Carnivora	0.259	0.036	0.000	0.000	0.171	0.054	0.678	6	O	52
207	†*Dipsalidictis*	Oxyaenidae	Oxyaeninae	Creodonta	1.337		0.000		0.406		0.407	1		
208	†*Oxyaena forcipata*	Oxyaenidae	Oxyaeninae	Creodonta	1.510		0.000		0.256		0.479	1	C	
209	†*Oxyaena gulo*	Oxyaenidae	Oxyaeninae	Creodonta	1.282	0.028	0.000	0.000	0.268	0.066	0.443	3	C	
210	†*Oxyaena intermedia*	Oxyaenidae	Oxyaeninae	Creodonta	1.260		0.000		0.281		0.435	1	C	
211	†*Palaeonictis*	Oxyaenidae	Ambloctoninae	Creodonta	0.573		0.000		0.341		0.272	1	O	
212	†*Palaeonictis occidentalis*	Oxyaenidae	Ambloctoninae	Creodonta	0.665		0.000		0.291		0.309	1	O	74
213	†*Patriofelis ferox*	Oxyaenidae	Oxyaeninae	Creodonta	2.120		0.000		0.025		0.663	1	HC	73
214	*Prionodon linsang*	Prionodontidae	Prionodontidae	Carnivora	0.100		0.000		0.134		0.802	1	C	53
215	*Bassaricyon gabbii*	Procyonidae	Procyonidae	Carnivora	0.798		0.000		1.034		0.273	1	O	62,73
216	*Bassariscus astutus*	Procyonidae	Procyonidae	Carnivora	0.572		0.000		0.431		0.444	1	O	62
217	*Bassariscus sumichrasti*	Procyonidae	Procyonidae	Carnivora	0.771	0.060	0.000	0.000	0.447	0.029	0.390	2	O	62
218	*Nasua narica*	Procyonidae	Procyonidae	Carnivora	0.852		0.000		0.876		0.288	1	O	62
219	*Nasua nasua*	Procyonidae	Procyonidae	Carnivora	0.858		0.000		0.849		0.291	1	O	62,73
220	*Nasuella olivacea*	Procyonidae	Procyonidae	Carnivora	0.835	0.033	0.000	0.000	0.837	0.013	0.297	3	I	62
221	*Potos flavus*	Procyonidae	Procyonidae	Carnivora	0.746		0.000					1	O	62,73
222	*Procyon cancrivorous*	Procyonidae	Procyonidae	Carnivora	0.660	0.029	0.000	0.000	0.560	0.014	0.386	2	O	54
223	*Procyon gloveralleni*	Procyonidae	Procyonidae	Carnivora	0.790		0.000		0.668		0.335	1		
224	*Procyon insularis*	Procyonidae	Procyonidae	Carnivora	0.845		0.000		0.564		0.346	1		
225	*Procyon lotor*	Procyonidae	Procyonidae	Carnivora	0.878		0.000		0.592		0.334	1	O	
226	*Procyon pygmaeus*	Procyonidae	Procyonidae	Carnivora	0.854	0.027	0.000	0.000	0.600	0.026	0.337	2	O	
227	†*Thylacinus cynocephalus*	Thylacinidae	Thylacinidae	Dasyuromorphia	1.539	0.050	2.149	0.174	0.271	0.025	0.113	2	HC	75
228	†*Agriotherium*	Ursidae	Ursidae	Carnivora	0.743		0.262		0.406		0.355	1	HC	76
229	*Ailuropoda melanoleuca*	Ursidae	Ursidae	Carnivora	0.946	0.030	0.621	0.027	0.660	0.035	0.235	5	He	60
230	*Helarctos malayanus*	Ursidae	Ursidae	Carnivora	1.189	0.079	0.727	0.097	0.586	0.081	0.216	3	O	60
231	†*Indarctos oregonensis*	Ursidae	Ursidae	Carnivora	0.881		0.390		0.419		0.310	1		
232	*Melursus ursinus*	Ursidae	Ursidae	Carnivora	1.168	0.128	0.524	0.102	0.640	0.012	0.227	2	O	60
233	*Tremarctos ornatus*	Ursidae	Ursidae	Carnivora	1.416	0.013	0.794	0.032	0.538	0.033	0.203	2	O	60
234	*Ursus americanus*	Ursidae	Ursidae	Carnivora	1.513	0.082	1.100	0.101	0.731	0.116	0.160	13	O	60
235	*Ursus arctos*	Ursidae	Ursidae	Carnivora	1.404	0.024	1.200	0.161	0.639	0.087	0.169	3	O	60
236	*Ursus maritimus*	Ursidae	Ursidae	Carnivora	1.220	0.014	0.799	0.085	0.598	0.050	0.207	4	HC	60
237	*Ursus thibetanus*	Ursidae	Ursidae	Carnivora	1.507	0.134	1.163	0.151	0.737	0.061	0.157	5	O	60
238	†*Amphicynodon leptorhynchus*	Ursidae/Ursoidea	Ursidae	Carnivora	0.513		0.162		0.543		0.387	1		
239	*Arctictis binturong*	Viverridae	Paradoxurinae	Carnivora	0.404	0.032	0.000	0.000	0.475	0.106	0.483	2	O	55
240	*Arctogalidia trivirgata*	Viverridae	Paradoxurinae	Carnivora	0.564	0.059	0.000	0.000	0.569	0.105	0.407	4	O	55
241	*Civettictis civetta*	Viverridae	Viverrinae	Carnivora	0.494	0.094	0.000	0.000	0.626	0.127	0.411	3	O	55
242	*Cynogale bennettii*	Viverridae	Paradoxurinae	Carnivora	0.562	0.006	0.000	0.000	1.153	0.229	0.297	2	O	55
243	*Genetta angolensis*	Viverridae	Genettinae	Carnivora	0.403	0.029	0.000	0.000	0.329	0.082	0.536	3	O	58
244	*Genetta genetta*	Viverridae	Genettinae	Carnivora	0.431	0.113	0.000	0.000	0.304	0.052	0.536	5	O	55,73
245	*Genetta maculata*	Viverridae	Genettinae	Carnivora	0.339	0.026	0.000	0.000	0.249	0.005	0.598	3	C	55
246	*Genetta servalina*	Viverridae	Genettinae	Carnivora	0.351	0.019	0.000	0.000	0.286	0.023	0.575	3	C	55
247	*Genetta thierryi*	Viverridae	Genettinae	Carnivora	0.321		0.000		0.327		0.570	1		
248	*Genetta tigrina*	Viverridae	Genettinae	Carnivora	0.243		0.000		0.336		0.602	1	C	55
249	*Genetta victoriae*	Viverridae	Genettinae	Carnivora	0.421	0.008	0.000	0.000	0.418	0.064	0.497	2		
250	*Hemigalus derbyanus*	Viverridae	Paradoxurinae	Carnivora	0.592	0.008	0.000	0.000	1.168	0.136	0.290	3	I	55
251	*Paguma larvata*	Viverridae	Paradoxurinae	Carnivora	0.314		0.000		0.477		0.515	1	O	55
252	*Paradoxurus hermaphroditus*	Viverridae	Paradoxurinae	Carnivora	0.294	0.049	0.000	0.000	0.625	0.134	0.476	4	O	55
253	*Paradoxurus zeylonensis*	Viverridae	Paradoxurinae	Carnivora	0.418		0.000		0.612		0.438	1	O	55
254	*Poiana richardsonii*	Viverridae	Genettinae	Carnivora	0.154	0.002	0.000	0.000	0.125	0.004	0.771	2		
255	*Viverra megaspila*	Viverridae	Viverrinae	Carnivora	0.250	0.011	0.000	0.000	0.401	0.037	0.571	2		
256	*Viverra tangalunga*	Viverridae	Viverrinae	Carnivora	0.369	0.041	0.000	0.000	0.597	0.060	0.457	3	O	55
257	*Viverra zibetha*	Viverridae	Viverrinae	Carnivora	0.262	0.016	0.000	0.000	0.477	0.057	0.536	4	O	55
258	*Viverricula indica*	Viverridae	Viverrinae	Carnivora	0.337	0.047	0.000	0.000	0.454	0.077	0.514	3	O	55

Supplementary Table S2. Regression results (RMA) of M2/M1 versus M3/M1 with indicated confidence intervals (CI).

	Slope	CI min	CI max	Intercept	CI min	CI max	r	p	n	Reference
Inhibitory cascade model	2.00			−1.00						13
Usag-1 WT-Het	0.77			−0.23						
Bmp7 WT-Het (not significant for M3/M1)	0.03			0.28						
Carnivora	0.59	0.487	0.742	−0.17	−0.222	−0.133	0.62	**0.000**	216	
Carnivora (with 3 molars; Canidae, Ursidae & Miacida	0.90	0.792	1.036	−0.29	−0.368	−0.229	0.96	**0.000**	43	
Canidae (with 3 molars)	0.45	0.376	0.515	−0.08	−0.104	−0.037	0.91	**0.000**	24	15
Canidae (excluding Otocyon)	0.48	0.412	0.537	−0.09	−0.119	−0.057	0.93	**0.000**	26	15
Canidae	0.90	0.619	1.358	−0.27	−0.483	−0.137	0.86	**0.000**	27	15
Ursidae	1.08	0.711	1.301	−0.52	−0.739	−0.040	0.93	**0.000**	11	
Craodonta	2.57	1.302	7.246	−2.31	−8.591	−0.720	0.39	**0.044**	27	
Hyaenodontidae	2.51	0.983	3.696	−1.91	−3.443	−0.077	0.63	**0.003**	20	
Dasyuromorphia	2.01	0.876	3.203	−1.31	−2.909	0.267	0.70	**0.001**	20	
Dasyuromorphia (excluding Sarcophilus)	2.32	1.274	3.665	−1.74	−3.571	−0.309	0.72	**0.000**	19	

Supplementary Table S3. Regression results (RMA) of M2/M1 versus tad/trd morphospace with indicated confidence intervals (CI).

	Slope	CI min	CI max	Intercept	CI min	CI max	r	p	n
Inhibitory cascade model	–			–					
Usag-1 WT-Het (not significant for tad/trd)	0.318			0.417					
Bmp7 WT-Het	0.513			0.285					
Carnivora	0.873	0.725	1.003	0.080	0.047	0.114	0.523	**0.000**	212
Canidae	0.757	0.596	0.854	0.062	0.022	0.126	0.911	**0.000**	32
Eupleridae	0.336	0.156	0.520	0.122	−0.023	0.179	0.918	**0.010**	6
Herpestidae	0.685	0.538	0.808	0.015	−0.058	0.102	0.783	**0.000**	29
Mephitidae	−4.341	−15.050	−1.781	2.150	1.227	5.454	−0.457	0.184	10
Mustelidae	3.905	2.400	5.140	−0.353	−0.593	−0.020	0.588	**0.000**	53
Procyonidae	2.042	−0.961	9.550	−0.940	−7.187	1.646	0.481	0.134	11
Ursidae	0.331	0.178	0.481	0.214	0.008	0.420	0.667	**0.025**	11
Viverridae	2.293	1.592	3.298	−0.362	−0.694	−0.057	0.719	**0.000**	20
Creodonta	−0.421	−1.419	−0.218	0.820	0.537	2.097	−0.195	0.349	25
Hyaenodontidae	0.603	0.359	2.097	−0.521	−2.476	−0.257	0.042	0.846	18
Oxyaenidae	−0.227	−0.642	−0.003	0.551	0.154	0.998	−0.716	0.071	7
Dasyuromorphia	−0.866	−2.824	−0.227	1.409	0.494	4.075	−0.214	0.365	20
Dasyuromorphia (excluding Sarcophilus)	−0.931	−1.370	−0.191	1.515	0.458	2.131	−0.496	**0.031**	19

Supplementary Table S4. Results of ANOVA–Tukey's test and phylogenetic ANOVA with a post-hoc Bonferroni–Holm test for differences in M2/M1 and tad/trd scores between dietary types (P value).

	M2/M1							tad/trd							Number of species for each dietary categories (HC/C/O/I)
**ANOVA–Tukey test**															
	Diet	HC vs C	HC vs O	HC vs I	C vs O	C vs I	O vs I	Diet	HC vs C	HC vs O	HC vs I	C vs O	C vs I	O vs I	
Carnivora	**0.000**	0.850	**0.000**	**0.000**	**0.000**	**0.000**	**0.002**	**0.000**	**0.018**	**0.000**	**0.000**	**0.000**	**0.000**	0.791	
Canidae	**0.000**	1.000	**0.008**	**0.000**	**0.004**	**0.000**	**0.001**	**0.000**	0.903	**0.001**	**0.000**	**0.003**	**0.000**	**0.000**	3/4/17/2
Eupleridae	0.194		0.444	0.182			0.424	0.058		0.095	0.053			0.439	1/0/3/2
Herpestidae	**0.036**						**0.036**	0.082						0.082	0/0/15/8
Hyaenidae								0.950						0.950	2/1/0/1
Mephitidae															0/0/6/0
Mustelidae	**0.024**	0.765	0.316		**0.032**			**0.000**	0.773	0.101		**0.000**			1/20/22/0
Procyonidae	0.607						0.607	0.506						0.506	0/0/9/1
Ursidae	0.601		0.682					0.565		0.625					2/0/6/0
Viverridae	0.061				0.299	0.053	0.170	**0.007**				0.133	**0.006**	**0.029**	0/3/12/1
Creodonta	**0.041**	0.280	**0.040**		0.439			**0.016**	0.057	**0.028**		0.747			11/7/4/0
Hyaenodontidae	0.321	0.292	0.899		0.780			**0.048**	0.129	0.088		0.794			10/4/2/0
Oxyaenidae	**0.004**	**0.023**	**0.004**		**0.014**			**0.004**	**0.005**	**0.004**		0.198			1/3/2/0
Dasyuromorphia	0.882	0.996	0.953	0.879	0.991	0.991	0.981	0.154	0.401	0.138	0.987	0.799	0.818	0.482	3/3/3/1
Dasyuromorphia (excluding Sarcophilus)	0.842	0.884	0.978	0.998	0.980	0.877	0.961	0.321	0.715	0.338	1.000	0.804	0.822	0.498	2/3/3/1
**Phylogenetic ANOVA test**															
	M2/M1							tad/trd							Number of species for each dietary categories (HC/C/O/I)
	Diet	HC vs C	HC vs O	HC vs I	C vs O	C vs I	O vs I	Diet	HC vs C	HC vs O	HC vs I	C vs O	C vs I	O vs I	
Carnivora	**0.001**	0.624	0.104	**0.010**	**0.020**	**0.006**	0.078	**0.001**	1.000	**0.006**	**0.006**	**0.006**	0.117	1.000	
Canidae	**0.001**	0.947	**0.020**	**0.006**	**0.006**	**0.006**	**0.006**	**0.001**	0.409	**0.006**	**0.006**	**0.006**	**0.006**	**0.006**	3/4/17/2
Eupleridae	0.175							**0.046**							1/0/3/2
Herpestidae	**0.017**						0.017	0.175						0.175	0/0/15/8
Hyaenidae	**0.001**							0.210							2/1/0/1
Mephitidae	–							–							0/0/6/0
Mustelidae	0.159							**0.036**							1/20/22/0
Procyonidae	0.591							0.478							0/0/9/1
Ursidae	**0.040**		0.040					0.104		0.104					2/0/6/0
Viverridae	0.098							**0.023**							0/3/12/1
Creodonta	0.349	0.604	0.537		0.604			**0.004**	**0.018**	**0.015**		0.426			9/7/4/0
Hyaenodontidae	0.776							**0.043**	0.123	0.123		0.470			8/4/2/0
Oxyaenidae	**0.033**							**0.037**							1/3/2/0
Dasyuromorphia	0.927							0.224							3/3/3/1
Dasyuromorphia (excluding Sarcophilus)	0.915							0.341							2/3/3/1

Supplementary Table S5. Measurements of Dasyuromorphia for additional tests.

Number	Species	Family	Subspecies etc.	Order	M3/M2	M3/M2SD	M4/M2	M4/M2SD	N	Diet	Reference
35	*Antechinus flavipes*	Dasyuridae	Dasyuridae	Dasyuromorphia	1.034	0.037	0.671	0.032	2		
36	*Antechinus godmani*	Dasyuridae	Dasyuridae	Dasyuromorphia	1.019	0.043	0.631	0.052	2		
37	*Antechinus hageni*	Dasyuridae	Dasyuridae	Dasyuromorphia	0.991	0.038	0.678	0.019	2		
38	*Antechinus leo*	Dasyuridae	Dasyuridae	Dasyuromorphia	1.057	0.082	0.690	0.037	2		
39	*Dasyuroides byrnei*	Dasyuridae	Dasyuridae	Dasyuromorphia	1.044	0.036	0.743	0.032	3		
40	*Dasyurus albopunctatus*	Dasyuridae	Dasyuridae	Dasyuromorphia	1.055	0.038	0.922	0.018	4	C	65
41	*Dasyurus geoffroii*	Dasyuridae	Dasyuridae	Dasyuromorphia	1.090	0.031	0.929	0.097	3	C	66,67
42	*Dasyurus hallucatus*	Dasyuridae	Dasyuridae	Dasyuromorphia	1.008	0.017	0.759	0.023	4		
43	*Dasyurus maclatus*	Dasyuridae	Dasyuridae	Dasyuromorphia	1.119	0.050	0.968	0.093	2	HC	66,67
44	*Dasyurus viverrinus*	Dasyuridae	Dasyuridae	Dasyuromorphia	1.101	0.038	0.948	0.056	4	C	66,68
45	*Murexia longicaudata*	Dasyuridae	Dasyuridae	Dasyuromorphia	1.030	0.025	0.717	0.024	3		
46	*Myoictis melas*	Dasyuridae	Dasyuridae	Dasyuromorphia	0.915	0.035	0.671	0.075	2		
47	*Neophascogale lorentzii*	Dasyuridae	Dasyuridae	Dasyuromorphia	1.042		0.802		1		
48	*Phascogale calura*	Dasyuridae	Dasyuridae	Dasyuromorphia	0.981	0.017	0.669	0.006	2	O	69
49	*Phascoloorex doriae*	Dasyuridae	Dasyuridae	Dasyuromorphia	1.008	0.008	0.785	0.064	2	O	70
50	*Planigale ingrami*	Dasyuridae	Dasyuridae	Dasyuromorphia	0.959	0.061	0.679	0.134	2	O	71
51	*Pseudantechinus macdonnellens*	Dasyuridae	Dasyuridae	Dasyuromorphia	1.014	0.032	0.728	0.021	2	I	72
52	*Sarcophilus harrisii*	Dasyuridae	Dasyuridae	Dasyuromorphia	1.105	0.013	1.028	0.052	3	HC	66,68
53	*Sminthopsis virginiae*	Dasyuridae	Dasyuridae	Dasyuromorphia	1.022	0.012	0.650	0.050	2		
227	*Thylacinus cynocephalus*	Thylacinidae	Thylacinidae	Dasyuromorphia	1.399	0.158	1.565	0.177	2	HC	75

Supplementary Table S6. Molar ratio and morphology of various genotypes in mice (genotypes: Uasg-1 and Bmp7).

Genotype	Mean M2/M1	Mean M3/M1	Mean tad/trd	SE M2/M1	SE M3/M1	SE tad/trd	n
HetHet	0.669	0.284	0.660	0.007	0.005	0.006	30
HetWT	0.656	0.278	0.626	0.010	0.006	0.009	16
WTHet	0.717	0.296	0.653	0.008	0.006	0.011	16
WTWT	0.678	0.295	0.633	0.015	0.007	0.011	10

Het: hetero-knockout
WT: wildtype

Supplementary Table S7. Result of the general linear model (GLM) to test the effects of Usag-1 and Bmp7 on relative molar sizes and M1 shape.

	Seq SS	Adj SS	Adj MS	*F*	*P*
**Effect on M2/M1**					
Usag-1	0.024	0.019	0.019	12.500	0.001
Bmp7	0.009	0.011	0.011	6.860	0.011
*Usag-1*Bmp7*	0.002	0.002	0.002	1.580	0.212
Residual error	0.106	0.106	0.002		
**Effect on M3/M1**					
Usag-1	0.003	0.003	0.003	4.900	0.030
Bmp7	0.000	0.000	0.000	0.300	0.589
*Usag-1*Bmp7*	0.000	0.000	0.000	0.160	0.693
Residual error	0.044	0.044	0.001		
**Effect on tad/trd**					
Usag-1	0.000	0.000	0.000	0.000	0.974
Bmp7	0.014	0.011	0.011	8.160	0.006
*Usag-1*Bmp7*	0.001	0.001	0.001	0.530	0.468
Residual error	0.094	0.094	0.001		

Supplementary Table S8. Log likelihood values, ω ratios, and parameters estimated using different models, and the likelihood ratio tests between two-ratio and one-ratio models.

		np	κ	l	ω0	ω1 (panda)	Likelihood ratio tests (two-ratio vs one-ratio)
pro-domain	free-ratio	23	4.71	−1705.953524			
	one-ratio	13	4.69	−1707.999081	0.0115		
	two-ratio	14	4.70	−1707.899477	0.0110	0.0161	2Δl=0.199208, p=0.6554
mature-domain	free-ratio	23	3.63	−962.466957			
(tree 1)	one-ratio	13	3.69	−977.078893	0.0198		
	two-ratio	14	3.68	−967.992451	0.0102	0.2473	2Δl=18.172884, p=0.00002017*
mature-domain	free-ratio	27	3.58	−971.118174			
(tree 2)	one-ratio	15	3.64	−987.760652	0.0188		
	two-ratio	16	3.63	−977.314257	0.0096	0.3486	2Δl=20.89299, p=0.000004857*

* significant (p<0.01)
np: number of parameters.
κ: transition/transversion rate ratio.
l: log likelihood value.

Supplementary Table S9. Results of homogeneity test of nonsynonymous/ synonymous change ratios.

pro-domain			
	N	S	p (two-tailed)
panda	2	16	
other carnivora	2	41	0.5743

mature-domain (tree 1)			
	N	S	p (two-tailed)
panda	7	5	
other carnivora	3	23	0.0047**

mature-domain (tree 2)			
	N	S	p (two-tailed)
panda	7	4	
other carnivora	3	43	0.00012**

* $p<0.05$
** $p<0.01$
